# Probing Nanoscale Features of Electrospun Amorphous Solid Dispersions Using Advanced Biophysical Analysis

**DOI:** 10.1021/acs.molpharmaceut.6c00342

**Published:** 2026-08-01

**Authors:** Luyao Wang, Abhishek Rajbanshi, Najet Mahmoudi, Evangelia Tsolaki, Thomas Zillhardt, Eleanor Hilton, Michael T. Cook, Gareth R. Williams

**Affiliations:** † UCL School of Pharmacy, 4919 University College London, 29-39 Brunswick Square, London WC1N 1AX, U.K.; ‡ Implexis Ltd., 37-63 Southampton Row, London WC1B 4DR, U.K.; § ISIS Pulsed Neutron and Muon Source, 15585 Rutherford Appleton Laboratory, Didcot OX11 0QX, U.K.; ∥ Diamond Light Source, Harwell Science and Innovation Campus, Fermi Ave, Didcot OX11 0DE, U.K.

**Keywords:** amorphous solid dispersion, electrospinning, aspirin, polycaprolactone, small-angle neutron scattering, drug-rich domain

## Abstract

Amorphous solid dispersions (ASDs) can significantly enhance the solubility of poorly soluble active pharmaceutical ingredients (APIs). However, the typical thermodynamic instability of ASDs is a significant barrier to commercial success, and understanding API recrystallization behavior is crucial for enhancing stability. While most techniques do not allow the effective exploration of early-stage crystallization, small-angle neutron scattering (SANS) has shown promise in the study of nanoscale drug domains in polymer–drug composite materials. In this study, aspirin was incorporated in electrospun polycaprolactone (PCL) fibers at loadings from 10–30% w/w. Electron microscopy revealed the fibers to have smooth surfaces and diameters of 2–3 μm. X-ray diffraction and differential scanning calorimetry revealed the presence of crystalline drug at 20 and 30% loadings. Infrared spectroscopy indicated the presence of intermolecular interactions between the drug and the polymer; however, these interactions were insufficient to completely inhibit drug crystallization when the drug was supersaturated. SANS revealed heterogeneous nanoscale structures within the fibers, characterized by concentration-dependent drug-rich domains in the polymeric matrix. Their precise solid-state nature (amorphous versus crystalline) could not be determined by SANS, though X-ray diffraction indicated the presence of crystalline material at higher drug loadings. The SANS data also indicated the growth of drug aggregates after exposure to water. In vitro drug release tests suggested that the size of the drug domains could influence drug release behavior. Overall, this study provides a number of new insights into the crystallization of APIs in electrospun ASDs, which can be used to help guide the future development of more effective medicines.

## Graphical abstract

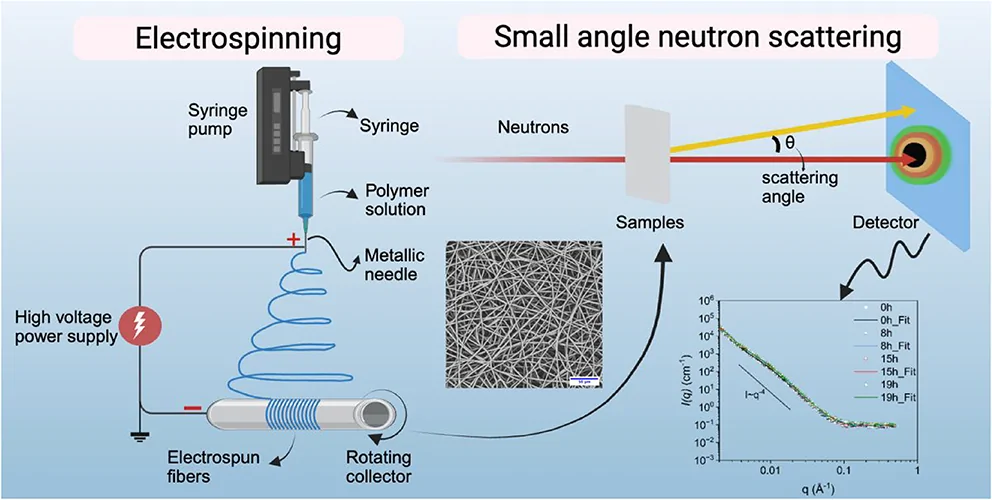

## 1 Introduction

The solubility and dissolution rate of active pharmaceutical ingredients (APIs) are intrinsically linked to their physical form. Currently, approximately 40% of marketed drugs are classified as low solubility by the biopharmaceutical classification system (BCS), as are nearly 90% of new drug candidates in development, presenting major challenges for therapeutic efficacy.^1^ A widely reported strategy to improve the solubility and dissolution rate of poorly water-soluble APIs is to formulate them as amorphous solid dispersions (ASDs), which require dispersing the API in a polymer carrier.^2–4^

Before introducing ASD formulations to the market, it is crucial to consider their physical stability.^5^ The physical instabilities associated with ASDs include amorphous–amorphous phase separation (AAPS) and/or the transformation of API from amorphous to crystalline form, which reduce the apparent solubility advantage of the amorphous form and consequently slow dissolution.^2,6^ In the AAPS phase, considered as an early stage of recrystallization, drug-rich domains form within the polymeric matrix where the drug was originally dispersed in molecular form.^2,7^ Subsequently, crystallization occurs due to the reduced polymer content within these domains. However, the impact of the AAPS phenomenon on the ASD performance remains incompletely understood.

Electrospinning is an emerging technique for manufacturing ASDs. Verreck et al.^8^ and Brewster et al.^9^ were among the first to report the preparation of amorphous drug–polymer nanofibers using electrospinning, demonstrating its ability to significantly enhance the dissolution of poorly soluble drugs. Electrospinning has a number of advantages.^10^ It directly converts polymer solutions into continuous micro- and nanofibers via electrical energy, offering higher energy efficiency and more efficient drying than other processes. The nanofibers’ high surface area and porosity are beneficial for generating higher dissolution rates than ASDs produced by other methods.^11^ The technique is scalable (to the ton scale) and can be undertaken under good manufacturing practice (GMP) conditions.^12–14^

The polymers used for electrospinning can be broadly classified into natural and synthetic polymers. Natural polymers, such as chitosan and cellulose, together with their derivatives, like hydroxypropyl methylcellulose (HPMC), are well-known for their biocompatibility and have a relatively low risk of an adverse immune reaction. However, their use in electrospinning can be limited by relatively weak mechanical properties and poor spinnability, often necessitating polymer blending.^15,16^ In contrast, synthetic polymers provide greater thermal stability, electrospinning processability, and tailorable mechanical properties.^15,17^ Among these, polycaprolactone (PCL), a semicrystalline and hydrophobic polymer, has been widely studied as a polymer carrier for electrospinning owing to its biocompatibility and biodegradability, and has been approved by the FDA.^18^ PCL exhibits limited water uptake and degradation, giving it a sustained drug release profile suitable for therapies requiring prolonged retention within the body.^19^ However, its low *T*_g_ (around −60 °C) allows substantial chain mobility at room temperature, which influences drug–polymer miscibility and the tendency of incorporated APIs to crystallize or phase separate.^20^

Small-angle neutron scattering (SANS) can noninvasively probe structures at length scales from around 1 nm to the near micron scale (about 600 nm).^21^ During a SANS experiment, a beam of neutrons is directed at a sample. When there is structural inhomogeneity within the sample, the neutrons mainly scatter from the nuclei of atoms within a small angle range and are collected on a 2D detector. The 1D-averaged intensity (*I*(*q*)) pattern is expressed as a function of the magnitude of the momentum transfer vector *q*, defined as *q* = [4π sin θ]/λ, where 2θ denotes the scattering angle, and λ represents the wavelength of the incident radiation,^22,23^ with *q* inversely related to the length scale detected within the sample: *d* = 2π/*q*. Therefore, the scattering intensity can reflect the structure and distribution of inhomogeneous features inside the sample,^22^ as shown in Figure 1. This renders SANS a suitable approach to study AAPS and early-stage recrystallization in ASDs.

**Figure 1:**
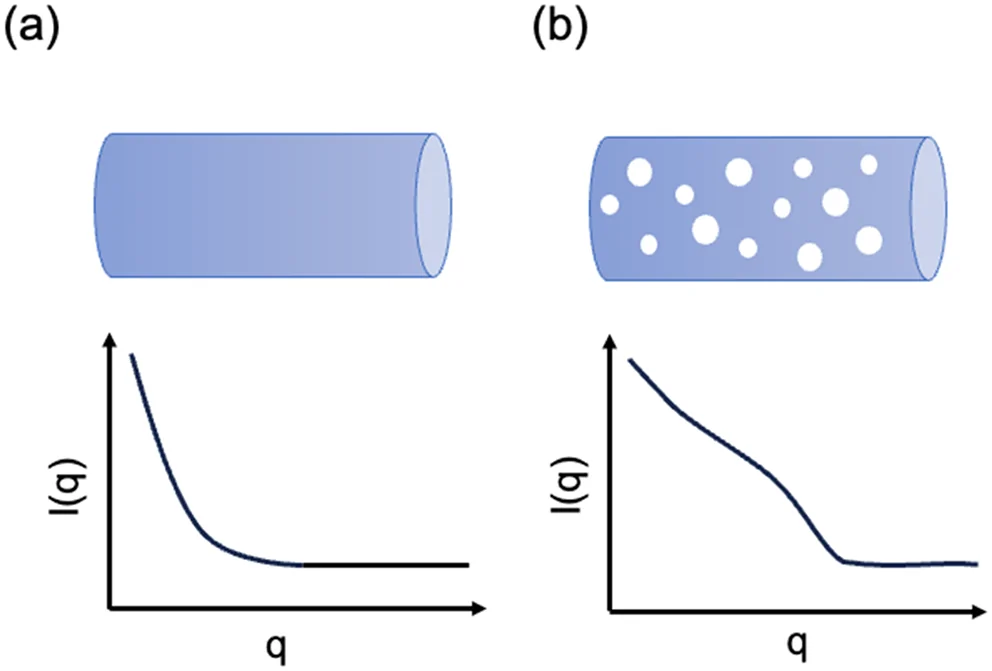
A schematic illustration showing differences in scattering between (a) homogeneous and (b) heterogeneous structures. In homogeneous systems, *I*(*q*) decreases smoothly with increasing *q*, indicating a uniform internal structure. In contrast, heterogeneous systems show a “shoulder” feature in the scattering profile, which arises from nonuniform domains.

Neutrons have high sensitivity for light elements and are thus ideally suited for the characterization of phases rich in hydrogen and other light elements.^23–25^ The scattering length density (SLD) is the key physical quantity in neutron scattering. The difference in SLD between distinct regions within a system, such as a polymer and its solvent, called contrast, contributes to the magnitude of SANS intensity, quantifying the inhomogeneous excess scattering length density fluctuations within a material, thereby creating structural contrast that enables SANS to “see” the nanoscale interfaces and distributions between them.^22,23,25,26^ This gives SANS notable advantages over other techniques for studying ASDs; the key advantages and disadvantages of each approach are summarized in Figure 2.^27–29^

**Figure 2:**
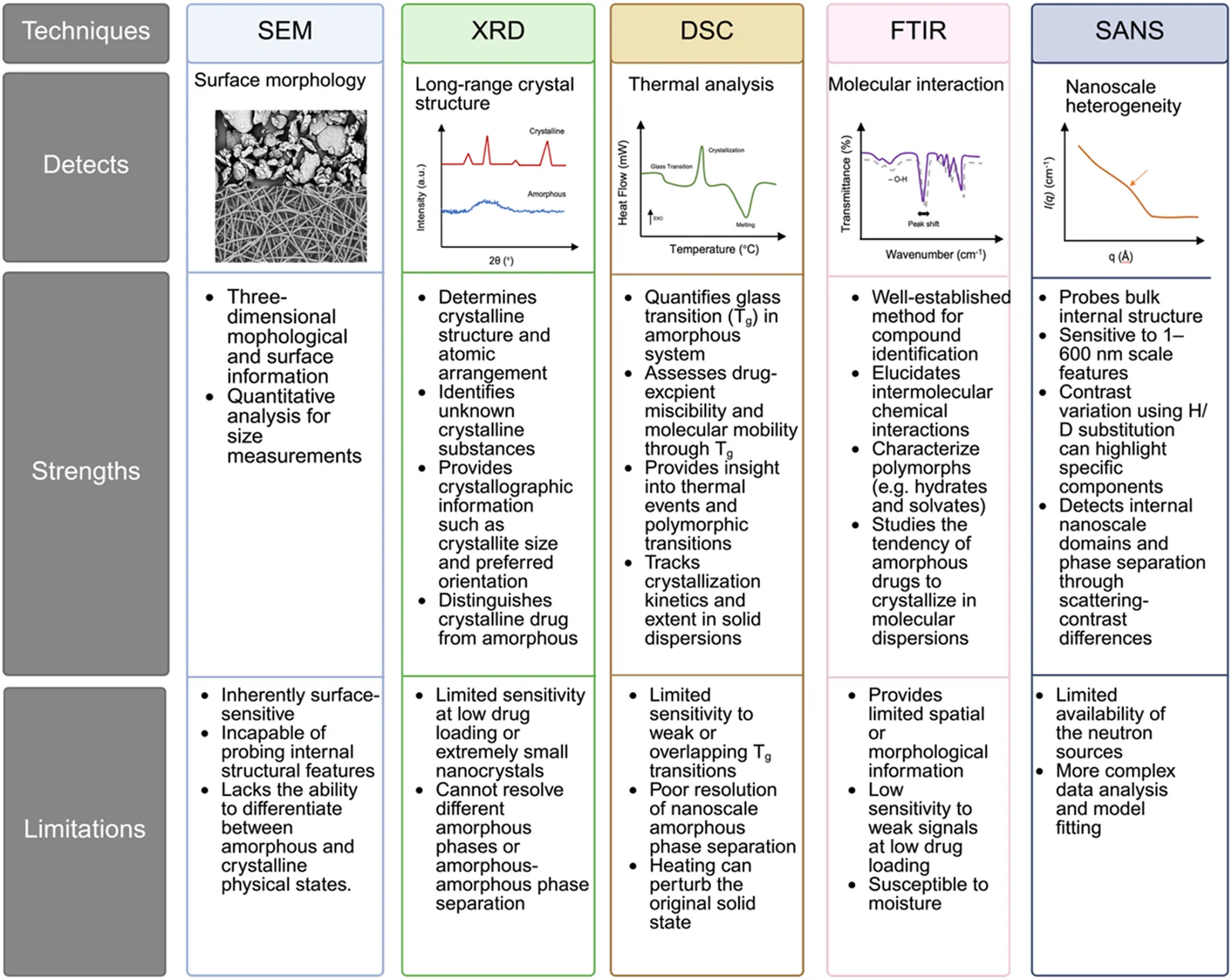
Schematic comparison of scanning electron microscopy (SEM), X-ray diffraction (XRD), differential scanning calorimetry (DSC), Fourier transform infrared spectroscopy (FTIR), and SANS for the characterization of ASDs. SEM provides information on surface morphology, XRD detects molecular/atomic order, DSC identifies thermal transitions, and FTIR reveals molecular interactions. In contrast, SANS is sensitive to internal nanoscale heterogeneity, enabling the detection of drug-rich domains formed during early-stage phase separation that are not readily accessible using conventional characterization techniques. Created in BioRender. Wang, L. (2026) https://BioRender.com/g2fyypf.

In this study, PCL fibers loaded with hydrogenated and deuterated aspirin were prepared by electrospinning, with the goal to probe the nanostructure of drug domains within these fibers. Aspirin, a classic nonsteroidal anti-inflammatory drug (NSAID), was used as a representative API, and a deuterated derivative was synthesized in-house. The fabricated fiber membranes were characterized by scanning electron microscopy, X-ray diffraction, thermal analysis, and vibrational spectroscopy. SANS was also conducted under both dry and wet conditions to study the physical form of the changes upon wetting. Finally, dissolution test studies were performed.

## 2 Materials and Methods

### 2.1 Materials

Polycaprolactone (PCL, Mn = 80,000 g/mol), aspirin, 1,1,1,3,3,3-hexafluoro-2-propanol (HFIP, ≥99%), ethanol (≥99.8%), deuterated acetic anhydride, and phosphate-buffered saline tablets (PBS; pH = 7.4) were purchased from Sigma-Aldrich (UK). Salicylic acid (≥99%) was obtained from BDH Chemicals Ltd. (UK). Sulfuric acid was purchased from Merck KGaA (Germany). All materials were of analytical grade and used without further purification.

### 2.2 Synthesis of Deuterated Aspirin (Aspirin-D)

Salicylic acid (3.7 g, 0.03 mol, 1 equiv) was added to a flask with deuterated acetic anhydride (5 mL, 0.05 mol, 2 equiv), followed by 5 drops of sulfuric acid with stirring. The reaction was heated to 80 °C for 15 min, then cooled to 0 °C. Upon precipitation, water (40 mL) was added, and the solids were collected by vacuum filtration. The solids were washed with cold water and dried under vacuum, then recrystallized from ethanol and poured into cold water to precipitate. The resulting colorless crystals were collected by vacuum filtration and dried by rotary evaporation to give aspirin-d_3_. The nuclear magnetic resonance (NMR) spectroscopy results shown in Figure S1 (Supporting Information) confirm successful synthesis.

### 2.3 Electrospinning of Aspirin and PCL Solutions

Solutions at a concentration of 12% w/v PCL were prepared in HFIP. The solutions were stirred using a magnetic stirrer (Cimarec i, Thermo Scientific, UK) at a rotational speed of 200 rpm overnight until all of the polymer was dissolved. For drug-loaded solutions, aspirin and deuterated aspirin at concentrations of 10, 20, and 30% (w/w, drug to polymer) were added to each solution, and the mixtures were stirred for 24 h until a homogeneous solution was obtained.

The electrospinning equipment used was a TL-BM-300 instrument, purchased from Tong Li Tech Co., Ltd. (China). The polymer solutions were loaded into a 5 mL plastic syringe and attached to a syringe pump (KDS100, KD Scientific, USA) to control the flow rate of the solution at a constant rate of 2 mL/h. The syringe was connected to a metal spinneret (21 gauge, 0.8 mm internal diameter, Nordson EFD, UK) through a polytetrafluoroethylene tube. The metal spinneret was attached to the positive electrode of a high-voltage power supply in the electrospinning equipment. The negative electrode was connected to a rotating mandrel collector with a diameter of 8 cm, covered with baking paper, and spinning at a speed of 500 rpm. A moving raster was employed to move the needle over a distance of 50 mm along the mandrel during the spinning process. A negative voltage of −3 kV was applied to the mandrel, and a positive voltage between 5 and 6 kV to the spinneret. The spinneret tip-to-collector distance was 15 cm. The relative humidity was between 30 and 40%, and the temperature was between 19 and 23 °C.

### 2.4 Scanning Electron Microscopy

The morphology of the fibers was observed using a benchtop scanning electron microscope (SEM; Phenom Prox, Thermo Fisher Scientific, USA). Prior to imaging, the samples were sputter-coated with gold using a Quorum 150R S Plus sputter coater at 20 mA for 60 s. The images were taken at an excitation voltage of 10 kV. The diameter of the fibers was measured from the SEM images on the basis of 100 randomly selected fibers, using the ImageJ software (National Institutes of Health, USA). The data obtained were reported as mean ± standard deviation (SD) and visualized as distribution graphs. Statistical analysis using one-way ANOVA followed by Tukey’s multiple comparisons test was performed in GraphPad Prism.

### 2.5 X-ray Diffraction

X-ray diffraction (XRD) patterns of the raw materials and fibers were collected on a MiniFlex 600 diffractometer (Rigaku, Japan), supplied with Cu–Kα radiation (λ = 1.5418 Å) at 40 kV and 15 mA. Samples were scanned over the 2θ range 3–50° at a speed of 5° min^–1^ (step = 0.02°). CIF files were obtained from the Cambridge Structural Database (CSD; CCDC 1986363). Predicted diffraction patterns were generated using the XRDlicious platform.^30^

### 2.6 Differential Scanning Calorimetry

Differential scanning calorimetry (DSC) was conducted using a Q2000 calorimeter (TA Instruments, USA). Approx. five mg of sample was placed in Tzero aluminum pans with pinholed Tzero aluminum lids (TA Instruments, USA). Samples were heated from 25 to 200 °C at a ramp rate of 10 °C/min. Nitrogen gas was purged through the instrument at a flow rate of 50 mL/min throughout the measurement.

### 2.7 Simultaneous XRD–DSC

Simultaneous XRD–DSC was performed following a methodology previously reported by Pang et al.^31^ All measurements were performed on the Joint Engineering, Environment and Processing (JEEP) Beamline I12 at the Diamond Light Source.^32^ A modified Q20 DSC (TA Instruments, USA) equipped with a refrigerated cooling system (RCS; TA Instruments, USA) was mounted on the beamline sample stage. Apertures were machined into the RCS and furnace assemblies, with entry openings of 5 mm (RCS) and 3 mm (furnace) and exit openings of 10 mm (RCS) and 5 mm (furnace). The instrument was precisely aligned such that the monochromatic X-ray beam (0.5 × 0.5 mm, λ = 0.234 Å) traversed the apertures of the DSC cell during heating. A Pilatus detector was positioned 1.9 m downstream to capture the diffraction patterns. Prior to data collection, the wavelength and sample-to-detector distance were calibrated using cerium dioxide (CeO_2_).^33^ Two masks were used to exclude pixels corresponding to interfaces between CdTe crystals and malfunctioning pixels (obtained in advance of the experiment). Image intensity was also rescaled with regard to the synchrotron ring current, by dividing the set point of 300 mA by the exact value recorded at the time of acquisition.

For the XRD–DSC experiments, approximately 5–10 mg of pure drug or electrospun fibers at 10 and 30% loadings were placed into Tzero aluminum pans (TA Instruments, USA) fitted with pinholed hermetic lids. The pans were then placed inside the modified DSC furnace and heated from 40 to 250 °C at a rate of 10 °C/min under a nitrogen purge. Thermal data were collected using TA Advantage and analyzed with TA Universal Analysis. Diffraction patterns were collected using the Diamond Generic Data Acquisition (GDA) system, with each exposure lasting 5 s and separated by a 12 s interval. The two-dimensional Pilatus detector frames were subsequently processed in the DAWN Science Workbench, where appropriate masking was applied before azimuthal integration to generate one-dimensional diffraction profiles.

### 2.8 Fourier Transform Infrared Spectroscopy

Fourier transform infrared (FTIR) spectra were obtained by using a Spectrum 100 spectrometer (PerkinElmer, USA). Data were collected over the wavenumber range from 4000 to 550 cm^–1^, with a resolution of 1 cm^–1^ and 8 scans obtained per sample.

### 2.9 Small-Angle Neutron Scattering

Small-angle neutron scattering (SANS) was conducted using the SANS 2D instrument at the ISIS Neutron and Muon Source (STFC, Rutherford Appleton Laboratory, UK). The beamline was configured with a collimation of 12 m, a beam size of 8 mm with a neutron wavelength range of 1.75–12.5 Å in the time-of-flight mode, and two detectors placed 5 and 12 m from the sample. This configuration covered a scattering wavevector (*q*) range of 0.0015 to 0.5 Å^–1^, corresponding to a size of approximately 12 to 4000 Å^–1^. For each measurement, rectangular samples (ca. 2 × 2 cm) were cut from the electrospun membranes, folded in half twice to form four layers, then sealed in aluminum foil to protect them from environmental exposure. Two samples were prepared from the same electrospun membrane: one was used directly for SANS measurement in the dry state, while the other was soaked in 200 μL of D_2_O, which was applied directly onto the fiber within the aluminum envelope. The soaked samples were measured at predefined time intervals (0, 8, 15, and 19 h) to observe structural evolution over time. All measurements were conducted at room temperature. The SANS data was processed using Mantid Workbench.^34^ Raw 2D data were corrected (incident neutron wavelength distribution, detector efficiency, measured transmission, and sample volume), radially averaged, scaled to absolute intensity using a standard polymer, merged, and background subtracted. The 1D reduced data corresponds to the coherent elastic differential scattering cross-section [*∂∑*/*∂Ω*(q)], commonly referred to as intensity, *I* [cm^–1^]. The reduced SANS data was analyzed in SasView (version 5, https://www.sasview.org/) using shape-independent models.

### 2.10 In Vitro Drug Release Studies

Drug release studies were conducted by immersing 10–40 mg of drug-loaded fibers into 20 mL of PBS. The setup was kept in a shaker incubator at 37 °C for 48 h. Two mL aliquots were periodically withdrawn, and the release media were replenished with an equal amount of fresh preheated PBS to keep the volume constant. Because aspirin gradually hydrolyzes to salicylic acid in PBS,^35^ both unhydrolyzed aspirin and its hydrolysis product (salicylic acid) were quantified in the release medium to accurately determine the amount of aspirin released. UV spectrophotometric measurements were performed at 278 nm for unhydrolyzed aspirin and at 296 nm for salicylic acid. The concentration of aspirin in each aliquot was determined using calibration curves established in PBS. The extinction coefficient for each drug was obtained from the slope of the Lambert–Beer plots. The mass of hydrolyzed aspirin was calculated with eq 1. The percentage of the released drug was calculated by eq 2. All release studies were performed under sink conditions. The test was repeated three times for each formulation, and the results are reported as mean ± SD.

1 $$t\mathrm{he}\;\mathrm{mass}\;\mathrm{of}\;\mathrm{hydrolyzed}\;\mathrm{aspirin}\;\left(\mathrm{\mu g}\right)=\frac{\mathrm{mass}\;\mathrm{of}\;\mathrm{salicylic}\;\mathrm{acid}\;\left(\mu g\right)}{\mathrm{Mw}\;\mathrm{of}\;\mathrm{salicylic}\;\mathrm{acid}\;(138.12\;g/\mathrm{mol})}\mathrm{\times Mw}\;\mathrm{of}\;\mathrm{aspirin}\;(180.16\;g/\mathrm{mol})$$

2 $$c\mathrm{umulative}\;\mathrm{release}\;\mathrm{of}\;\mathrm{aspirin}\;\left(\%\right)=\frac{\mathrm{mass}\;\mathrm{of}\;\mathrm{aspirin}\;\mathrm{released}}{\mathrm{mass}\;\mathrm{of}\;\mathrm{aspirin}\;\mathrm{loaded}\;\mathrm{in}\;\mathrm{fibers}}\times 100\%$$

## 3 Results and Discussion

### 3.1 Morphology and Size Distribution

SEM images and size distributions of the fibers are shown in Figure 3. All of the samples are characterized by cylindrical fibers without any beads-on-string phenomena, suggesting the formation of homogeneous solid dispersions. The drug-loaded fibers have no visible drug crystals on the surface. Fibers with 10 and 20% drug loading exhibit smooth surfaces, whereas those with 30% loading display a degree of roughness, with some wrinkling observable along the fiber axis. The mean diameter of the fibers is summarized in Figure 4. Compared with the blank fiber (2.05 ± 0.01 μm), drug-loaded fibers exhibit an increase in diameter. For aspirin-loaded fibers, the average fiber diameter increases significantly to 2.25 ± 0.05 μm at 10% loading (*p* < 0.01), and to 2.58 ± 0.07 and 2.81 ± 0.07 μm at 20 and 30% loading, respectively (*p* < 0.001). For aspirin-D-loaded fibers, a similar trend is observed, with fiber diameters of 2.54 ± 0.06, 2.82 ± 0.06, and 2.98 ± 0.06 μm at 10, 20, and 30% loading, respectively, all of which were significantly larger than that of the blank fibers (*p* < 0.001). A greater w/w percentage of drug incorporation, therefore, leads to a significant increase in diameter, as shown in Figure 4. This occurs due to the increase in the solute mass expelled from the spinneret per unit time, which is consistent with previous studies.^36,37^

**Figure 3:**
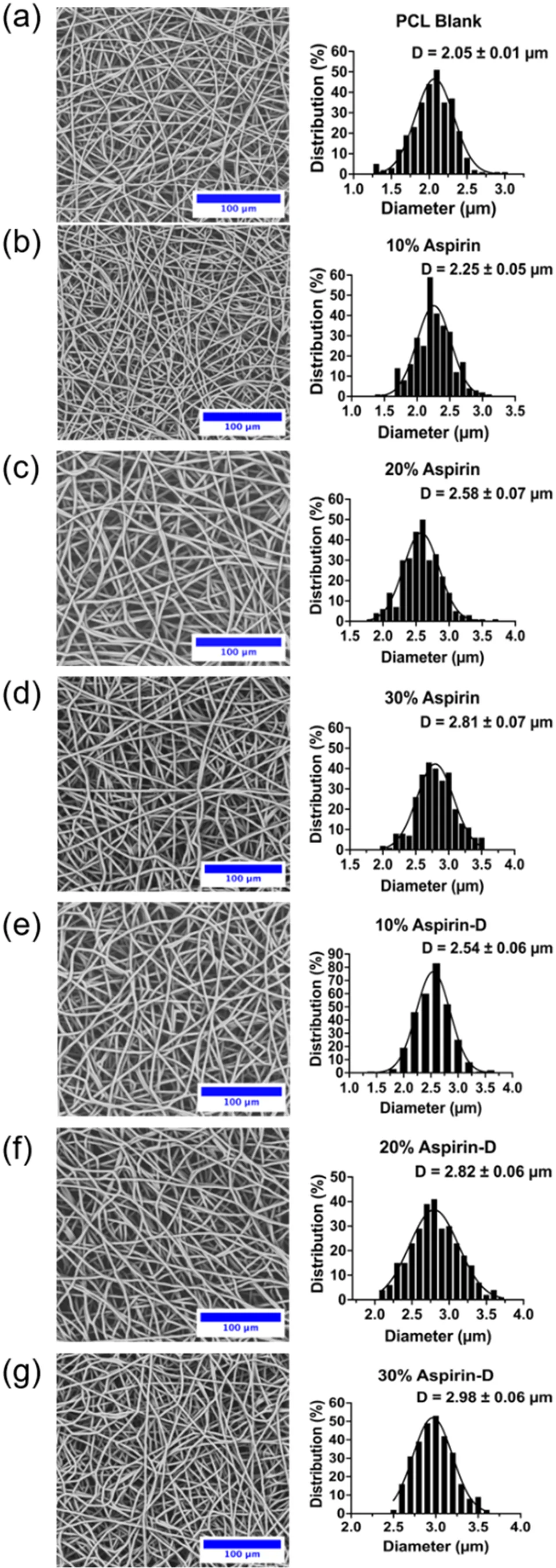
SEM images and size distributions of the fibers: (a) PCL blank, (b) 10% aspirin, (c) 20% aspirin, (d) 30% aspirin, (e) 10% aspirin-D, (f) 20% aspirin-D, and (g) 30% aspirin-D.

**Figure 4:**
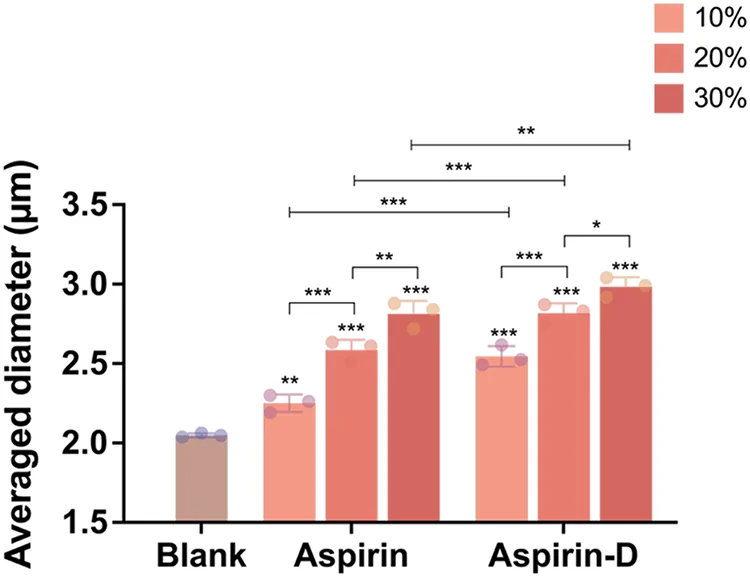
Diameters of the electrospun fibers. Data are presented as mean ± SD (*n* = 3). Statistical analysis vs blank fibers using one-way ANOVA followed by Dunnett’s multiple comparison test. For drug-loaded fibers, the effects of drug type and concentration were determined by two-way ANOVA, followed by Tukey’s multiple comparisons test. Statistical significance is indicated in the figure (*p < 0.05; **p < 0.01; ***p < 0.001).

At the same % w/w drug loading, the aspirin-D formulations have larger diameters than those made with aspirin. The reasons for this are not fully clear, but it is known that deuteration can alter noncovalent interactions through changes in molecular polarity, polarizability, and molecular volume.^38^ Therefore, the larger diameters observed for aspirin-D-loaded fibers may reflect subtle isotope-dependent differences in drug–polymer/drug–solvent interactions, which could influence polymer/drug mixing and solution spinnability. The diameter of fibers is also influenced by several parameters during electrospinning, including applied voltage, temperature, and humidity.^39^

### 3.2 XRD Data

XRD was conducted to examine the physical form of PCL, aspirin, aspirin-D, the drug-free fiber, and drug-loaded fibers (Figure 5). The XRD patterns of aspirin and aspirin-D exhibit distinct Bragg reflections at 7.7, 15.6, 23.4, and 27.1° (Figure 5a), consistent with the Form I polymorph.^40^ The diffraction peak positions of aspirin and aspirin-D remain the same, but the relative intensities of the peaks change. This can be attributed to the different morphologies of the particles and preferred orientation.^41^ PCL displays two characteristic diffraction peaks at 22.1 and 24.6° (Figure 5b),^42^ revealing its semicrystalline nature. The PCL blank fibers show the same two distinctive peaks of PCL but slightly shifted to lower angles. This demonstrates that the stretching during electrospinning allows the ordered arrangement and crystallization of the polymer chains.^43^

**Figure 5:**
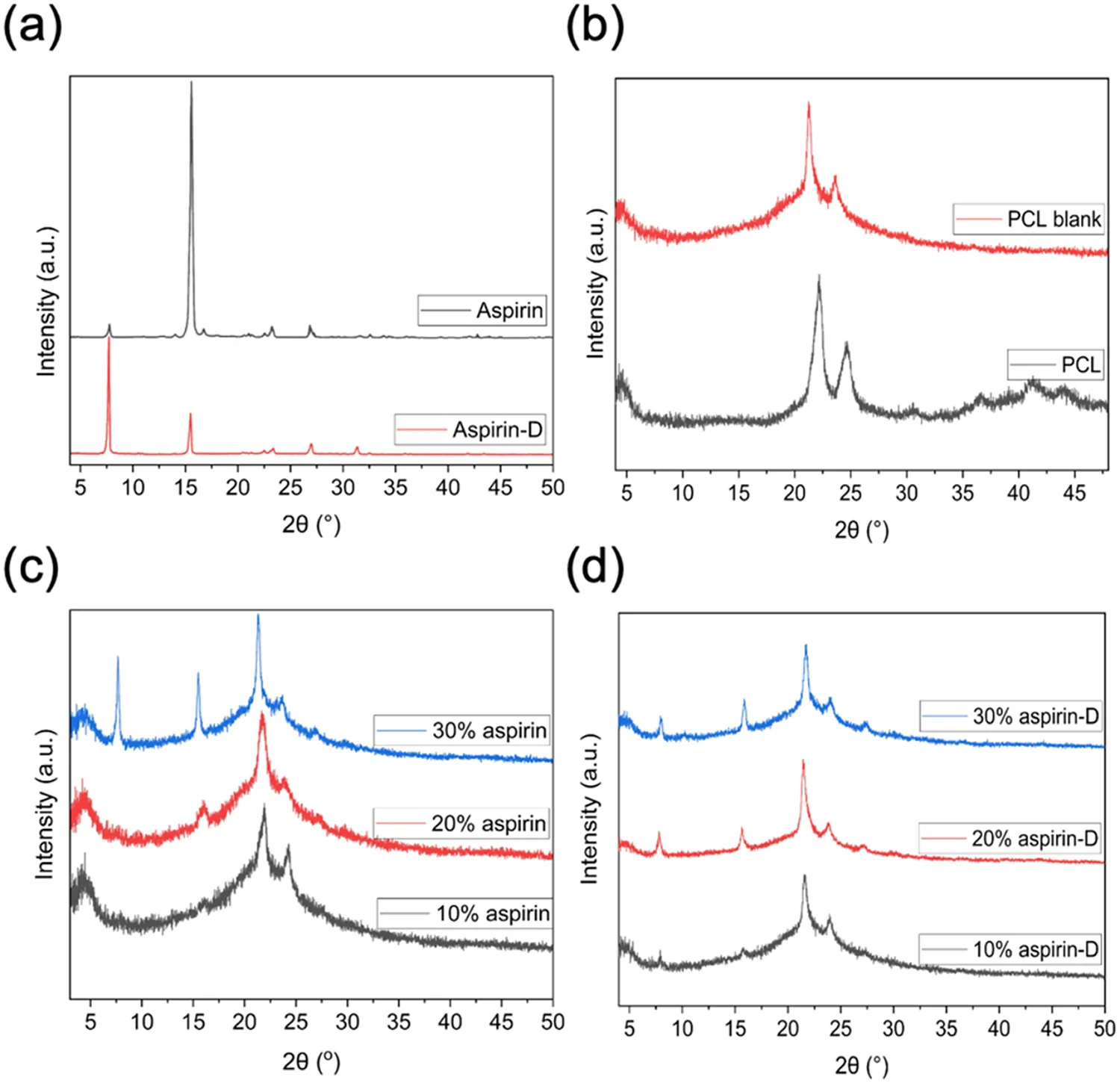
XRD patterns: (a) aspirin and aspirin-D; (b) PCL and PCL blank fibers; and the fibers containing (c) aspirin, and (d) aspirin-D.

XRD patterns of the drug-loaded fibers are presented in Figure 5c,d. All of the fibers show similar XRD patterns to the blank PCL fibers, presenting two sharp peaks at 21.3 and 23.6°. When fibers were prepared at 10% drug loading, the absence of sharp diffraction peaks related to aspirin indicates the API is mostly present in the amorphous state.^44,45^ The characteristic diffraction peaks of aspirin (at 2θ = 7.7 and 15.6°) become more obvious when the aspirin content is increased to 20 and 30%. Similar observations are seen with aspirin-D (Figure 5d). In all cases, there is clearly some crystalline drug present in the formulations.

The average crystallite sizes of the fibers were calculated by the Debye–Scherrer equation (*D* = *K*λ/[β cos θ], where *D* is the average size of the ordered domains, λ is the wavelength of the X-ray radiation, θ is the diffraction angle, and β is the full width at half-maximum (fwhm)^46^). The crystallite size estimated from the (002) reflection located at 2θ = 15.6 °C is ca. 5.6, 16.8, 7.1, and 12.0 nm for 20% aspirin, 30% aspirin, 20% aspirin-D, and 30% aspirin, respectively. These values represent approximate coherence length rather than a real size, but clearly indicate the presence of nanoscale crystalline domains and that the size of these increases with drug loading.^46^

### 3.3 DSC Analysis

DSC traces of the raw materials and electrospun fibers are presented in Figure 6. The melting onset (*T*_onset_) and peak temperature (*T*_peak_) are shown in Table 1. Aspirin and aspirin-D exhibit *T*_onset_ at 141.1 and 134.5 °C and *T*_peak_ at 143.0 and 138.1 °C, respectively. This confirms their crystalline nature.^47,48^ The DSC thermogram of PCL shows a sharp endothermic peak at 57.5 °C (*T*_onset_ = 51.6 °C) corresponding to its melting point (Figure 6a).^42,49^ The PCL blank fiber exhibits a slightly higher melting temperature than that of pure PCL, at 59.1 °C (*T*_onset_ = 55.6 °C).

**Figure 6:**
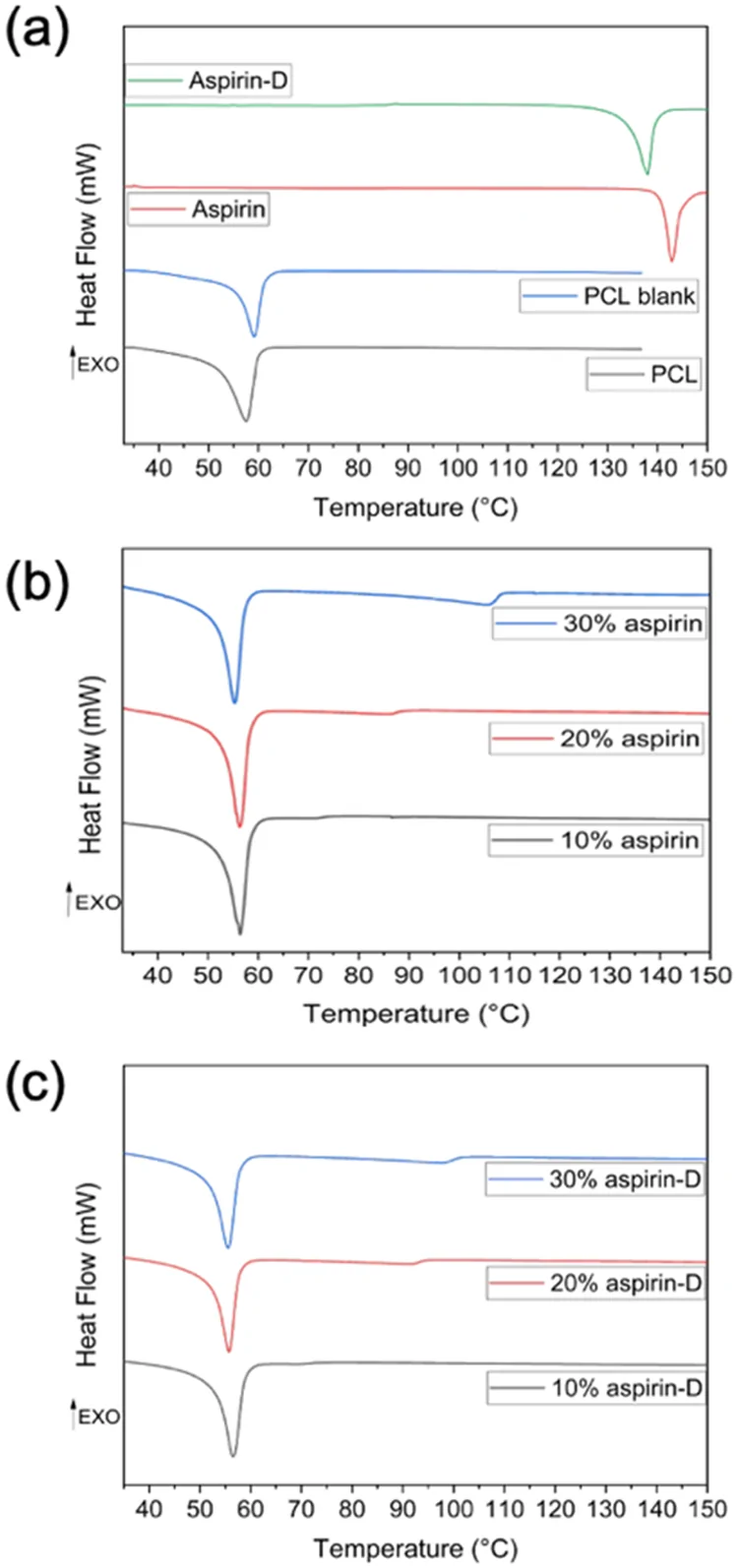
DSC curves of (a) aspirin-D, aspirin, PCL, and PCL blank fibers; and fibers loaded with (b) aspirin, and (c) aspirin-D.

**Table 1:** Summary of Onset and Peak Temperature Obtained from DSC Thermograms of all Samples

	*T*_onset_ (°C)		*T*_peak_ (°C)	
sample	PCL	API	PCL	API
PCL	51.6		57.5	
PCL blank	55.6		59.1	
aspirin		141.1		143.0
aspirin-D		134.5		138.1
10% aspirin	52.5		56.5	
20% aspirin	53.1	70.0	56.4	85.9
30% aspirin	51.1	84.4	54.6	102.9
10% aspirin-D	53.1		56.6	
20% aspirin-D	52.4	68.6	55.7	91.3
30% aspirin-D	51.7	79.8	55.6	97.7

For the aspirin-loaded fibers (10, 20, and 30%), the melting endotherm of PCL is shifted to *T*_peak_ = 56.5, 56.4, and 54.6 °C, respectively. Similarly, aspirin-D loading at 10, 20, and 30% leads to PCL melting at *T*_peak_ = 56.6, 55.7, and 55.6 °C, respectively. This phenomenon may arise from interactions between aspirin or aspirin-D and PCL, indicating their strong miscibility. For the 10% samples, no endothermic peak other than that of PCL was observed, indicating that the aspirin was largely present in an amorphous state, consistent with the XRD results (Figure 5c,d). A broad endothermic event was observed at 20% loading, with *T*_peak_ of 85.9 °C for aspirin and 91.3 °C for aspirin-D. When the loading increased to 30%, the *T*_peak_ increased to 102.9 and 97.7 °C for aspirin and aspirin-D, respectively. This is markedly lower than the melting point of the pure drug. However, the XRD results (Figure 5c,d) clearly indicate the presence of crystalline drug at both 20 and 30% loading. Hence, it is believed that this endothermic event must be related to the melting of crystalline aspirin within the PCL matrix. The dramatic reduction in temperature could perhaps be ascribed to the depression of the melting point in an impure sample. Alternatively, the drug particles could potentially dissolve into the molten polymer upon heating, resulting in an endothermic feature below the pure drug’s melting point. Simultaneous XRD–DSC measurements were performed to investigate which of these explanations is correct.

### 3.4 Simultaneous XRD–DSC

A contour plot was used to display the XRD patterns (with a color scale) alongside the DSC data. This representation enables straightforward tracking of variations in crystalline structure. The XRD–DSC data for pure aspirin are shown in Figure S2. The diffraction pattern shows three bright lines, indicating sharp diffraction peaks. This demonstrates that aspirin is crystalline, consistent with the laboratory XRD results (Figure 5a). The DSC data only shows one endothermic peak with an onset at 144 °C. The disappearance of the diffraction pattern at the peak of this endothermic peak (at 151 °C) indicates that the aspirin has completed melting at this point.

Figures 7 and 8 show the XRD–DSC results of 10 and 30% aspirin-loaded fibers, respectively. Both show an endothermic peak with onset at 51.3 and 50.4 °C, which is related to the melting of PCL. For 10% aspirin (Figure 7), the two distinct Bragg reflections in the XRD pattern disappear at 59.2 °C, suggesting the complete melting of PCL. No other diffraction peaks are present in the data, indicating that aspirin at 10% loading exists largely in the amorphous state, consistent with the laboratory XRD and DSC results (Figures 5c and 6b). The 30% aspirin-loaded fiber shows a second endothermic peak with onset at 87.6 °C and peak value at 103.2 °C. In contrast to 10% aspirin, some Bragg reflections are observed in the XRD pattern beyond 59.4 °C (after PCL melting). These correspond to crystalline aspirin within the fiber. When the temperature exceeds the peak of the second endothermic peak in the DSC curve, the aspirin Bragg reflections also disappear. This thus confirms the presence of crystalline aspirin, which melts at a temperature markedly lower than that of the pure drug.

**Figure 7:**
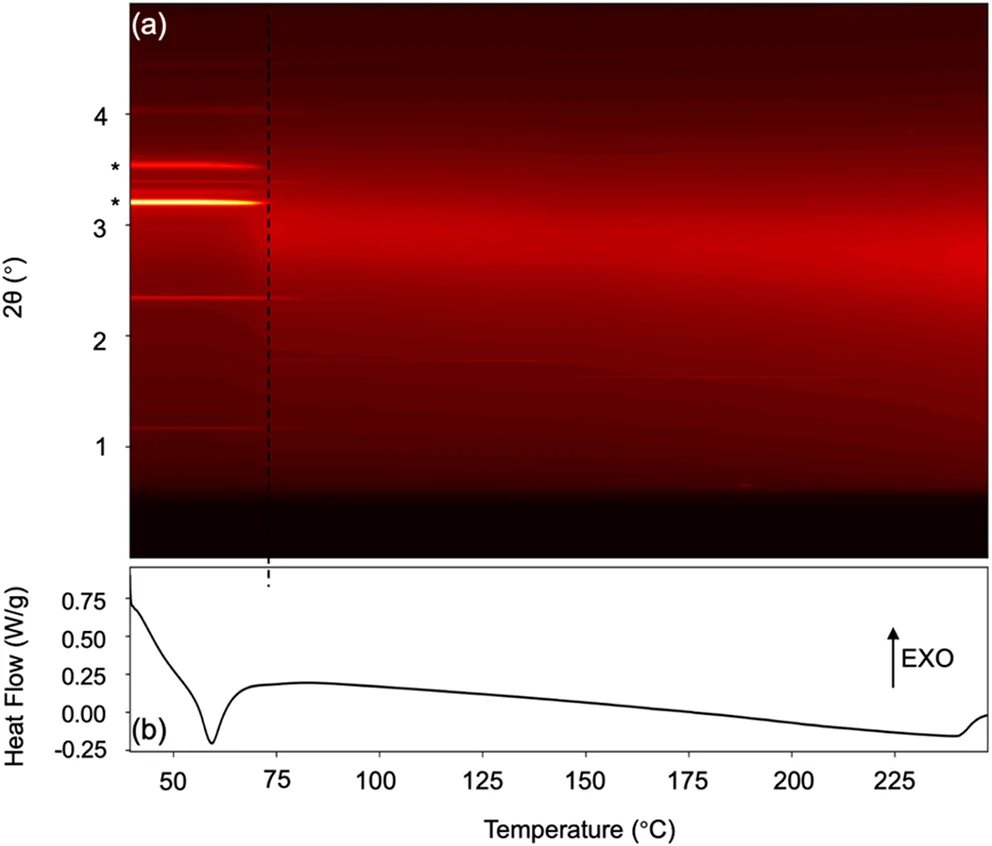
XRD–DSC data for 10% aspirin fibers. (a) A contour plot of XRD patterns (brighter colors denote stronger diffracted intensity) and (b) the corresponding DSC thermogram. Reflections marked with asterisks arise from PCL. All data are plotted as a function of temperature, and the upper and lower panels share the same temperature range on the *x*-axis.

**Figure 8:**
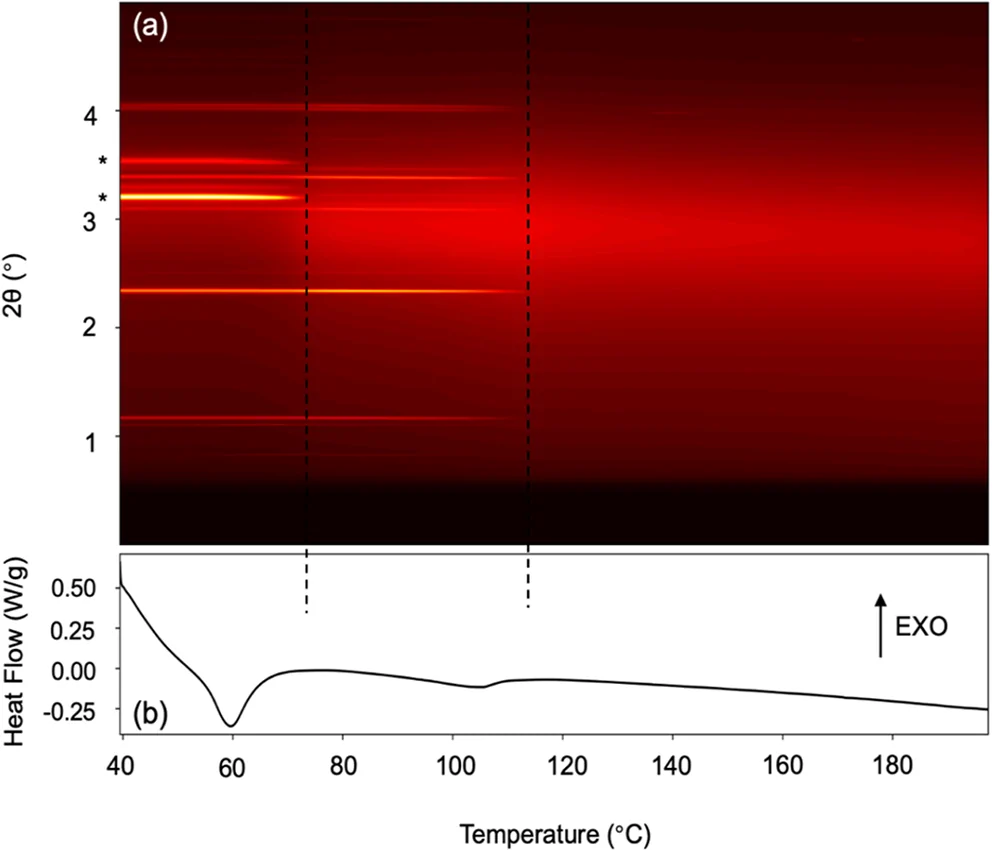
XRD–DSC data of 30% aspirin fibers. The upper and lower panels are aligned along the same temperature axis, showing (a) a heat map of XRD data with brighter colors indicating stronger diffracted intensity and (b) the corresponding DSC trace. Reflections marked * correspond to PCL.

### 3.5 FTIR Data

FTIR spectra of the raw materials are given in Figure 9. Aspirin has characteristic peaks at 2500–3500 cm^–1^ (C–H and O–H stretching), 1747 cm^–1^ (ester C=O stretching), 1680 cm^–1^ (carboxylic acid C=O stretching), and 1370 cm^–1^ (−CH_3_ symmetric bending).^50,51^ After deuteration, the characteristic C–H bending band originally observed around 1370 cm^–1^ disappears. Several new bands emerge at lower wavenumbers, which are attributed to the C–D deformation vibrations of the trideuteromethyl group.^52^ PCL shows signals at 2944 cm^–1^ (asymmetric CH_2_ stretching), 2868 cm^–1^ (symmetric CH_2_ stretching), 1724 cm^–1^ (C=O stretching), 1293 cm^–1^ (C–O and C–C stretching), and 1162 cm^–1^ (asymmetric C–O–C stretching).^49,53^ The electrospun PCL fiber has a very similar spectrum to raw PCL.

**Figure 9:**
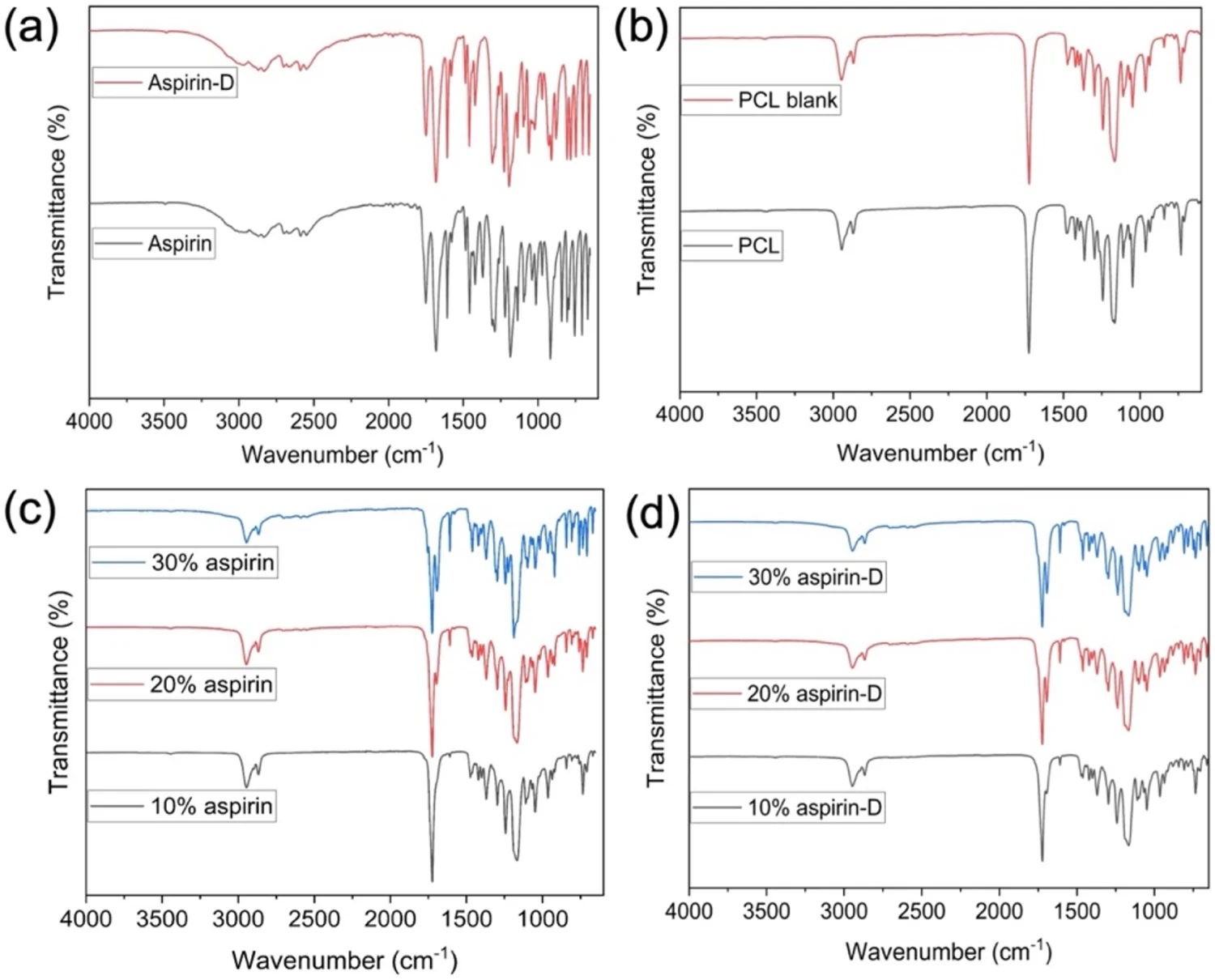
FTIR spectra of (a) aspirin-D and aspirin, (b) PCL and the blank fibers, and electrospun fibers with (c) aspirin and (d) aspirin-D.

The drug-loaded fibers (Figure 9c,d) show IR spectra similar to the PCL blank fibers in Figure 9b. However, with an increase in the drug loading, some characteristic peaks from aspirin or aspirin-D start to appear (e.g., at 2500–3000, 1690, and 1605 cm^–1^). In addition, the positions of some peaks are slightly shifted compared to the pure PCL spectra. For example, the C=O stretching band (1724 cm^–1^) from PCL is slightly shifted to a lower wavenumber, while the C=O stretching (1680 cm^–1^) from aspirin or aspirin-D is slightly shifted to a higher wavenumber. These observations may be attributed to the presence of intermolecular interactions between aspirin or aspirin-D and PCL, such as H-bonding.

### 3.6 Small-Angle Neutron Scattering

For the blank PCL fibers (Figure 10), the scattering intensity can be expressed by the Power-Law shape-independent form factor *I­(q) = A·q* ^*–n*^ *+ B*, where *A* is a scale factor, *B* represents the constant incoherent background (estimated from the limit of *I*(*q*) at high values of *q*), and n is the scattering exponent. In the high-*q* region, the scattering behavior is dominated by structural interference at length scales smaller than the size of the scattering objects. This typically results in a Power Law decay where the exponent n provides insight into the interfacial structure of the scattering objects.^54^ When *n* = 4, the scattering object has a sharp interface (smooth surface), which may be termed “Porod-type” scattering.^54,55^ When *n* falls within the range 3 < *n* < 4, the object shows surface fractals, and structures where 1 ≤ *n* ≤ 3 are defined as mass fractal-like aggregates.

**Figure 10:**
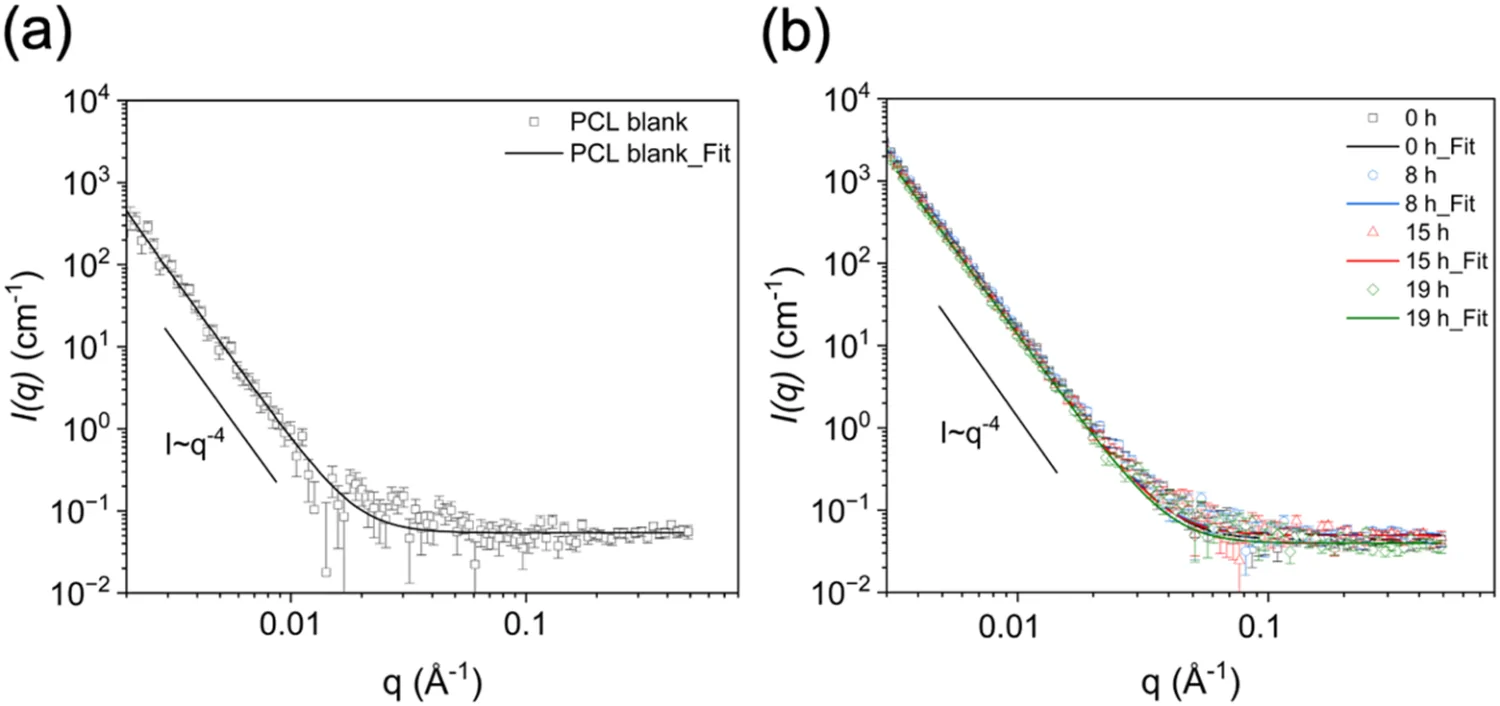
Experimental SANS data and the corresponding shape-independent Power-Law fits for the PCL blank fibers under (a) dry conditions and (b) after immersion in D_2_O for varied periods of time. The scattering intensity, *I­(q)*, is plotted as a function of the scattering vector, *q*, on logarithmic scale. Symbols represent experimental data, and solid lines represent model fits. The reference slope indicates *I*∼*q* ^–4^, corresponding to Porod-like scattering from smooth interfaces.

The scattering profiles of the drug-free fibers under both dry conditions (Figure 10a) and in D_2_O (Figure 10b) can be fitted with a ∼*q* ^–4^ decay function. No other scattering features are observed over 19 h of incubation, indicating that a homogeneous interior with a smooth surface is retained. The scattering profiles also suggest that the fibrous membrane remains structurally intact, without any structural changes potentially observable by SANS (e.g., surface erosion, internal microrupture, and water-filled pore formation^56^). Given that PCL is a hydrophobic material known to hydrolyze slowly in water, all of these observations are as anticipated.

SANS is sensitive to the SLD differences between different components. In the dry state, the SLD of both aspirin (2.49 × 10^–6^ Å^–2^) and aspirin-D (3.88 × 10^–6^ Å^–2^) is different from the PCL matrix (0.85 × 10^–6^ Å^–2^). To analyze the results, as per a previous study,^56^ the data was fitted to a Power Law model plus a Correlation Length model in the following functional form:

$$I\left(q\right)=\frac{A}{q^{n}}+\frac{C}{1+{\left(q\xi \right)}^{m}}+B$$

The first term describes the Porod scattering in the low-*q* (ca. < 5 × 10^–2^ Å^–1^) area, and the second term is a Lorentzian function describing fluctuations in structural composition in the high-*q* area, associated with small objects. *A* and *C* represent scale factors. In this model, factor *C* is proportional to the scattering intensity and is thus sensitive to the volume fraction and SLD contrast of the domain relative to the surrounding matrix. *B* is the incoherent background. Parameter *m* is the Lorentzian (high-*q*, > 1 × 10^–1^ Å^–1^) exponent, and *n* is the Porod (low-*q*) exponent. When *m* = 2, this functional form becomes the Lorentzian function. The exponent *m* is constrained to values ≤ 4 to ensure a physically realistic representation of finite-range structural correlations. The remaining parameter *ξ* is the average correlation length for the scattering objects. In drug-loaded systems, *ξ* may be related to the drug-rich domains. The second term of the model, characterized by parameters *C*, *ξ*, and *m*, enables the investigation of drug domain evolution. The interpretation of the SANS profiles is summarized schematically in Figure 11.

**Figure 11:**
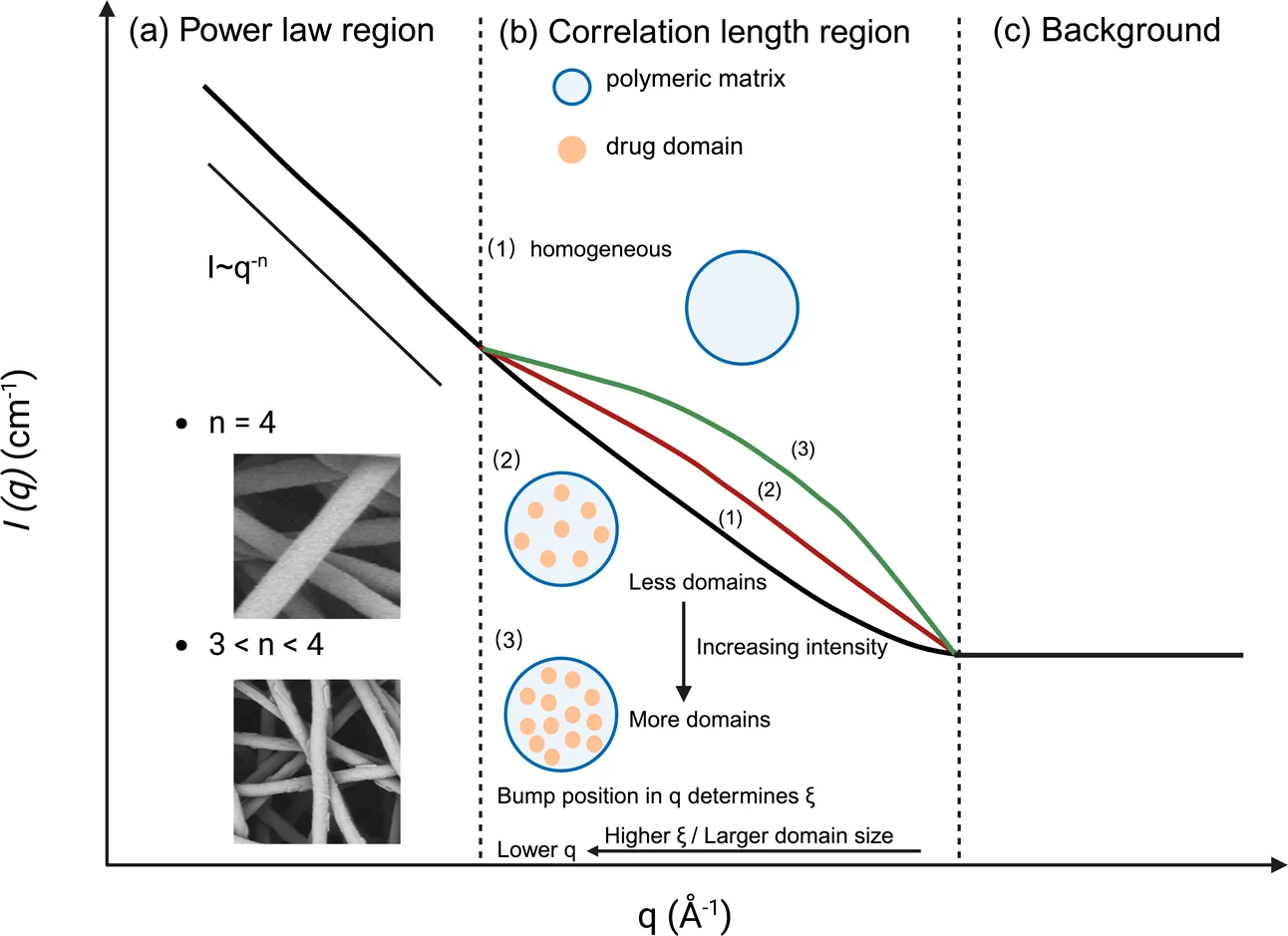
Schematic illustration of the physical meaning of key features obtained from SANS profiles fitted to a Power Law model plus a Correlation Length model. (a) In the Power Law region, the scattering intensity follows *I* ∼ *q^–n^*, where *n* describes the interfacial character of the fibrous membrane. *n* = 4 indicates Porod-type scattering from smooth interfaces, whereas 3 < *n* < 4 indicates surface-fractal scattering from rougher interfaces. (b) The Correlation Length region reflects nanoscale SLD fluctuations associated with drug-rich domains in the polymer matrix. The black curve labeled (1) represents a relatively homogeneous matrix with minimal nanoscale heterogeneity. The red curve labeled (2) represents the formation of drug-rich domains with a lower fitted *ξ*, and the green curve labeled (3) represents an increased number of drug-rich domains with a higher fitted *ξ*. The increase in scattering intensity reflects the greater volume fraction of domains, whereas the position of the scattering feature along q determines *ξ*, with a shift to lower q corresponding to larger domain size and higher *ξ*. (c) This region corresponds to the background contribution. Created in BioRender. Wang, L. (2026) https://BioRender.com/vr7aj9s.

Figure 12 shows model fitting to the dry state data. For fibers loaded with 10 or 20% aspirin, or aspirin-D, the scattering intensity in the low-q area can be fit to a ∼ *q* ^–4^ decay, indicating smooth fiber surfaces consistent with the SEM results (Figure 3b,c,e,f). However, with 30% aspirin and aspirin-D loaded fibers, the exponent *n* is found to be 3.5 and 3.7, respectively. This implies a partially rough interface with structural complexity on the nanoscale. These results are again consistent with the SEM observations (Figure 3d,g). The scale factor *C* and *ξ* for the scattering features at high-q values were also fitted (Figure 13). Aspirin-D exhibits a more pronounced SLD difference relative to the PCL matrix than aspirin, and thus a greater scale value (Figure 13a). As the drug loading rises, the *ξ* values also increase, indicating that the correlation length associated with drug-related inhomogeneous domains within the fiber matrix becomes larger (Figure 13b). This finding is consistent with the results from XRD (Figure 5c,d), DSC (Figure 6b,c), and FTIR (Figure 9c,d) characterization, the analysis of which revealed that characteristic peaks of the API appear as its w/w content increases.

**Figure 12:**
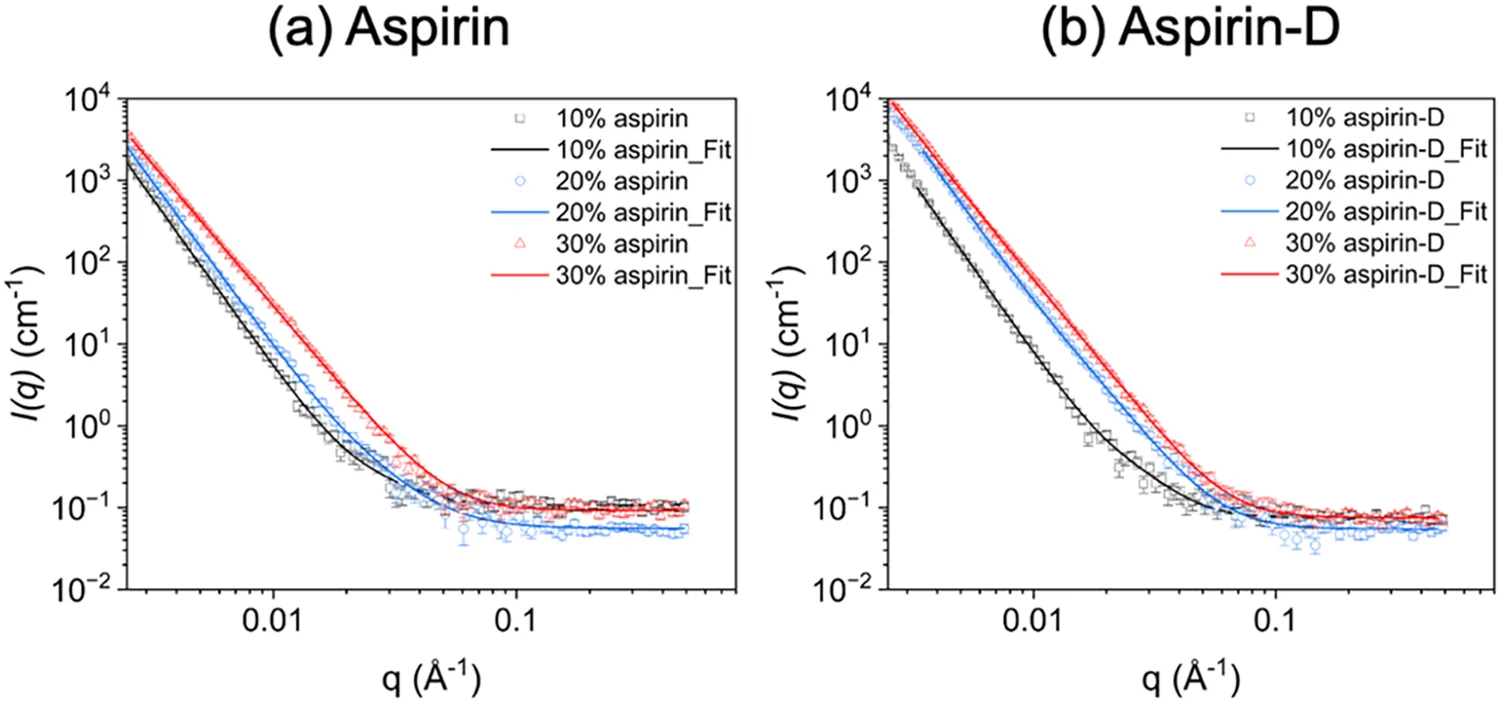
Experimental SANS data and the corresponding fits for fibers loaded with (a) aspirin and (b) aspirin-D in the dry state. Profiles are shown as *I­(q)* against *q* on log–log axes. Symbols indicate experimental data, and solid lines indicate a Power Law model plus a Correlation Length model fit.

**Figure 13:**
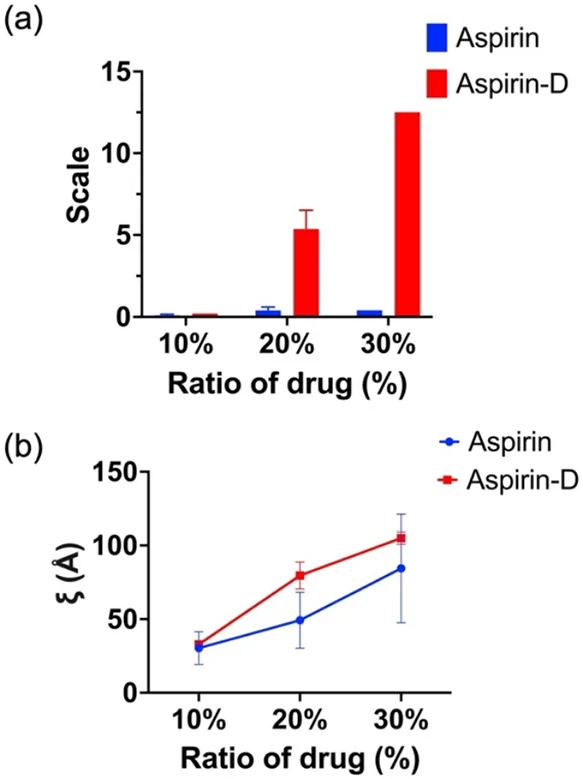
Fitted parameters extracted from dry-fiber SANS profiles. (a) Scale *C* and (b) correlation length *ξ* derived from Correlation Length model fitting of aspirin- and aspirin-D-loaded electrospun fibers. Data are shown as mean ± SD. The data of the fibers at 30% loading are fitted manually, so errors cannot be determined.

The addition of D_2_O to the drug-loaded fibers (Figure 14) leads to the rapid appearance of a scattering characteristic feature (a broad shoulder) at *q* ∼ 0.01 Å, which is more pronounced at higher drug loadings. This points to a drug-rich domain forming, though further analyses are required to confirm this hypothesis. The low-q region can be fitted to the Power Law model, indicating smooth surface features. The scattering from the 30% aspirin-loaded formulations in D_2_O can be fitted to a ∼ *q* ^4^ decay, while in the dry state, *n* < 4. This may be attributed to a smooth surface being formed after contact with D_2_O as the drug on the fiber surface dissolved.

**Figure 14:**
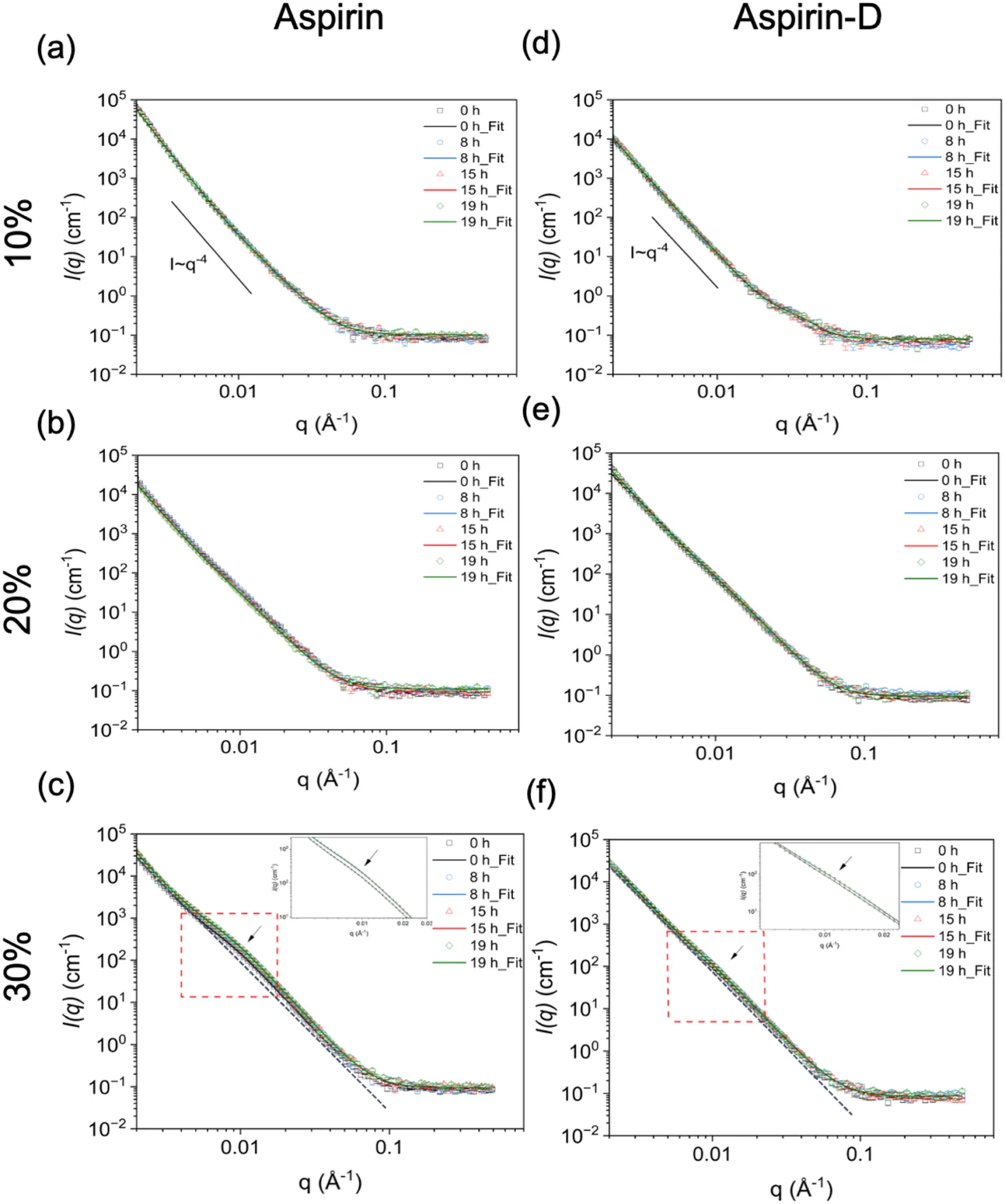
SANS profiles of drug-loaded electrospun fibers after immersion in D_2_O. Experimental SANS data and the corresponding Power Law model and Correlation Length model fits are shown for (a) 10% aspirin; (b) 20% aspirin; (c) 30% aspirin; (d) 10% aspirin-D; (e) 20% aspirin-D; and (f) 30% aspirin-D-loaded fibers after immersion in D_2_O for 0, 8, 15, and 19 h. The profiles are plotted as *I­(q)* versus *q* on log–log axes. Arrows highlight the mid-q shoulder in the Correlation Length region, which is related to drug-rich domains within the polymer matrix.

To confirm that the scattering characteristics arise from drug-rich domains, the data for aspirin and aspirin-D can be compared. The values of ξ and C for aspirin and aspirin-D-loaded fibers are given in Figure 15. The SLD of D_2_O is 6.39 × 10^–6^ Å^–2^, which is very different from the SLD of both aspirin and PCL, but closer to the SLD of aspirin-D. Upon incubation in D_2_O, the scattering contrast between aspirin and the surrounding medium increases due to the large SLD difference, thereby allowing SANS to detect nanostructured inhomogeneities within the polymeric matrix. In contrast, aspirin-D exhibits a reduced scattering intensity from drug-rich domains due to the diminished contrast with the surrounding environment, making these domains less distinguishable in the SANS profiles. Consequently, the large difference in SLD between aspirin and PCL effectively “amplifies” the signals from the drug. This amplification not only increases the overall scattering intensity (larger scale values) but also captures larger-scale structural fluctuations within the system, resulting in a larger fitted ξ value. Hence, the values of scale factor and ξ found with aspirin-D are both lower than those with aspirin (Figure 15). This confirms that the scattering characteristics seen by SANS in the mid-*q* region (∼0.01 Å) originate from drug-rich domains, supporting the previous hypothesis.

**Figure 15:**
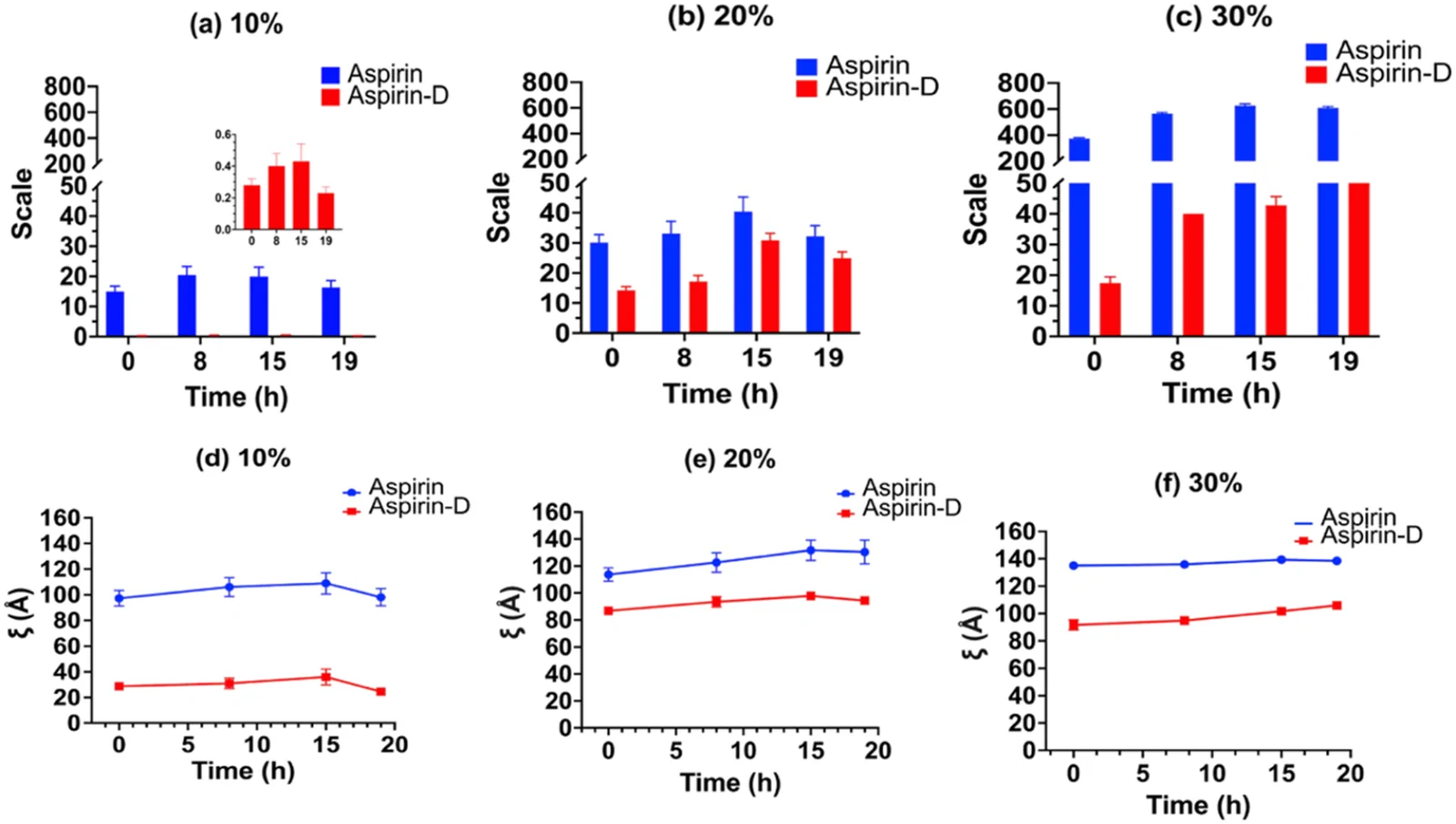
Values of scale and *ξ* derived from Correlation Length model fitting of SANS profiles for drug-loaded electrospun fibers incubated in D_2_O at room temperature over 19 h. The value of the scale factor *C* is shown for (a) 10% (inset: enlarged view of aspirin-D), (b) 20%, and (c) 30% w/w drug loading, and the evolution of *ξ* for (d) 10%, (e) 20%, and (f) 30% w/w drug content. The data are shown as mean ± SD.

The API molecules tend to aggregate within the fibers upon contact with water. After contact with D_2_O, a difference in SLD between drug aggregates and the polymer matrix is generated, allowing SANS to resolve these aggregates. Both the ξ and scale factor *C* increase within 15 h, as shown in Figure 15, reflecting the dynamic evolution of drug aggregation within the fiber matrix. At 15 h, the *ξ* values for aspirin-loaded fibers at 10, 20, and 30% are around 109, 132, and 139 Å, respectively. For the aspirin-D-loaded fibers, the respective *ξ* values are about 36, 98, and 101 Å. This phenomenon arises because after contact with D_2_O, the water molecules penetrate the fiber interior, inducing the formation of drug aggregates and resulting in an increase in ξ. At the same time, the high contrast between aspirin or aspirin-D and D_2_O leads to an overall increase in signal intensity and an increased scale *C* value. The simultaneous increase in *ξ* and scale *C* marks the transition of the drug molecules from a relatively uniform dispersed state to an aggregated state within the polymer matrix, comprising the early stage of phase separation. An increase in drug loading leads to a simultaneous rise in both *ξ* and *C* values, demonstrating a concentration-dependent tendency toward phase separation, whereby higher drug loading promotes the formation and growth of domains. Notably, the dramatic increase in scale and ξ observed during the initial contact with D_2_O (at 0 h), particularly at high drug loading (at 30%), may indicate the instantaneous formation of large-scale drug-enriched domains and water channels. This could potentially lead to a burst release of the drug from the fibers.

After 15 h, the *ξ* and scale factor *C* values of fibers containing 10 and 20% API exhibited a slight downward trend. Meanwhile, the values of the 30% fibers remained almost unchanged or showed a slight increase. The decrease in *ξ* and factor *C* may be because the drug aggregates gradually dissolve and diffuse into the D_2_O/fiber matrix. According to a previous study,^56^ as the drug-related structures gradually disappear and become uniformly dispersed within the fibers, the SLD contrast between the drug and its surrounding environment diminishes, eventually leading to the disappearance of the characteristic scattering features in SANS profiles. However, even at 19 h, the *ξ* and *C* values for all fibers are high, indicating that aggregates remain present within the fibers. This is probably due to the final solution having reached saturation, which inhibits further dissolution. The theoretical drug concentration in D_2_O, assuming complete dissolution, would be 25, 38, and 68 mg/mL for aspirin at 10, 20, and 30%, and 24, 55.5, and 77 mg/mL for aspirin-D at 10, 20, and 30%, respectively. The solubility of aspirin in water at room temperature is about 3 mg/mL.^57^ This phenomenon may also relate to the formation of water-filled pores after dissolving the drug aggregates. These water-filled pores preserve the structural heterogeneity and maintain scattering contrast, thereby continuing to contribute to the scattering signal.

### 3.7 In Vitro Drug Release Studies

The results of in vitro release studies for all fibrous membranes are shown in Figure 16. PCL exhibits extremely slow degradation rates in water and resistance to erosion attributed to its semicrystalline properties.^58^ Therefore, the drug release mechanism of PCL electrospun fibers is primarily diffusion-controlled.^59^

**Figure 16:**
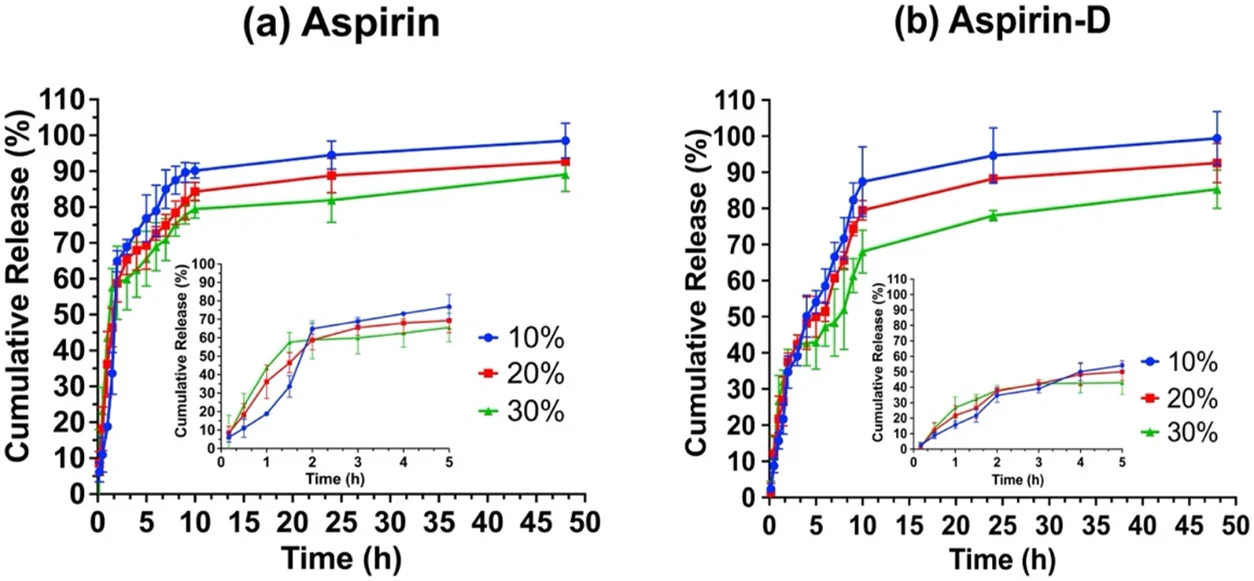
In vitro cumulative release of (a) aspirin and (b) aspirin-D from electrospun PCL fibers into PBS at 37 °C. All data are shown as mean ± SD.

All of the samples show an initial burst release within 1.5 h (10% aspirin: 34%, 20% aspirin: 46%, 30% aspirin: 58%, 10% aspirin-D: 22%, 20% aspirin-D: 27%, 30% aspirin-D: 32%). Higher drug loadings lead to a more pronounced burst release, which is due to the rapid release of drug molecules close to the surface. The 30% aspirin system shows the most notable burst release. This may also be related to the presence of the drug at the fiber surfaces (surface roughness in SEM (Figure 3d)) and the formation of numerous pore structures, as indicated by the evolution of *ξ* and scale *C* values in the SANS data (Figure 15). These findings show that fibers at 30% loading may immediately form extensive pore structures upon initial contact with water, potentially further accelerating drug dissolution and resulting in a sudden release.

Subsequently, the release profile enters a diffusion-controlled phase characterized by a relatively constant and slow release. In this phase, the drug release process proceeds via a two-step mechanism: initial dissolution of the embedded drug within the polymer matrix, followed by diffusion of the dissolved molecules into the release medium,^20^ as shown in Figure 17. Both release profiles show that a higher drug concentration is associated with slower release rates in this phase (after around 1.5 h), consistent with the previous study.^56^

**Figure 17:**
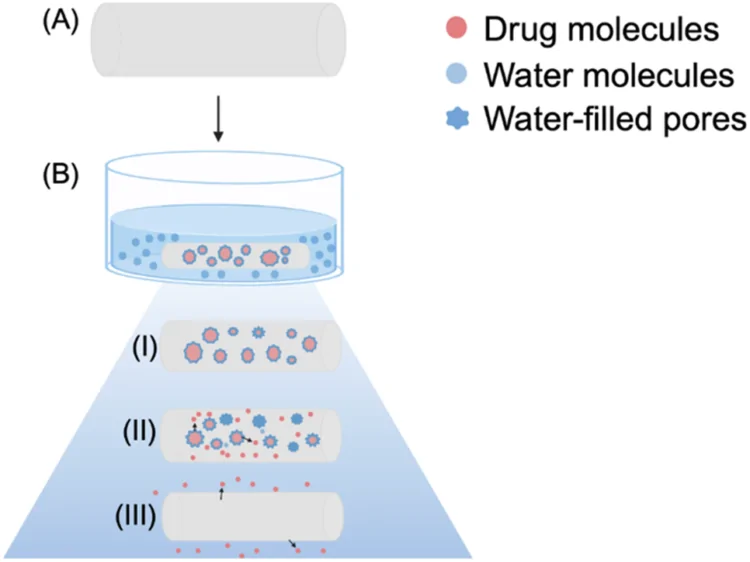
Illustration of the release of API from the PCL fibers. (A) Fibers in the dry state. The drug molecules are dispersed in the polymeric matrix. (B) When the fibers are soaked in water, (I) water molecules permeate into the polymer matrix and reach the drug aggregates within the polymer, which may form upon contact with water or pre-exist within the fibers at high loadings. (II) These drug aggregates are then dissolved. Smaller drug aggregates dissolve more rapidly than larger aggregates. Dissolved drug molecules enter the surrounding matrix from the aggregates through relaxation of the polymer matrix, and (III) drug molecules dispersed in the matrix diffuse into the release medium, toward an evenly distribution inside the fiber. Created with BioRender. com.

To compare the release behavior independently of the initial drug loading, the profiles of the released drug mass are shown in Figure S3. During the initial 2 h, the cumulative mass of drug released is similar regardless of the drug loading, at ∼1 mg. After the initial burst release, the cumulative mass released from the 30% fibers increases more slowly than in the other systems, such that at the end of the experiment, for both aspirin and aspirin-D, the 30% fibers reach the lowest overall released mass. The percentage- and mass-based release profiles showed consistent loading-dependent behavior. This suggests that the fraction of the drug available for diffusion decreased with increasing loading. Created in BioRender. Wang, L. (2026) https://BioRender.com/ow252lh.

The rate of drug release appears to be affected by the dissolution of drug aggregates within the fiber matrix. SANS analysis revealed the formation of drug-rich aggregates within the fiber matrix. As the drug loading increased, both the size and number of these aggregates were found to increase significantly. Such structural organization within the fibers is expected to have an impact on the release kinetics.^60^ Water molecules continuously penetrate the fibers and dissolve the drug molecules that are encapsulated deep within the polymer, and these dissolved drugs diffuse into the surrounding medium. However, due to the intrinsic hydrophobicity of the PCL, water penetration into the fiber interior and the dissolution of the drug occur at a slow rate.^61^ In addition, PCL exhibits semicrystalline properties, featuring structurally dense crystalline regions with compact stacking.^58^ As a result, larger and more densely packed aggregates impose significant barriers to the dissolution step (as illustrated in Figure 17B­(II)). This hindered dissolution becomes the primary rate-limiting factor, slowing down the overall release process. Previous studies have also demonstrated that fibers with larger diameters tend to exhibit a slower release rate, which may be attributed to the longer diffusion pathways and the lower surface area-to-volume ratio.^62,63^ The mean diameters of the fibers increase with drug loading (Figure 4), which may also contribute to the slower drug release observed at higher loadings. The release rate of aspirin-D is lower than that of aspirin, which may be attributed to the larger diameters of these fibers compared to their aspirin-loaded analogues at the same loading.

To further explore these possible contributions, cumulative drug release at 8 h was plotted against the fiber diameter and SANS-derived *ξ* values (Figure S4). For both aspirin- and aspirin-D-loaded fibers, cumulative release showed a stronger apparent negative correlation with fiber diameter than with *ξ*. This suggests that the fiber diameter, and therefore the effective diffusion path length through the hydrophobic PCL matrix, is dominant in controlling the loading-dependent release behavior. The weaker correlations with *ξ* indicate that nanoscale drug-rich domain characteristics may also contribute. Owing to the limited number of formulations, these relationships are interpreted qualitatively.

## 4 Conclusions

In this study, we successfully used SANS technology to capture the nanostructural dynamic evolution of electrospun aspirin-PCL ASDs during incubation in D_2_O. SEM results showed the successful preparation of microscale cylindrical fibers without bead-on-string morphology. Fibers with high drug loading exhibit wrinkling on their surface due to the likely presence of some drug aggregates. XRD indicates that the API is largely present in an amorphous state at a 10% w/w drug load, while the crystalline API is clearly visible at 20 and 30% drug loading. Simultaneous XRD–DSC analysis confirmed that an endothermic event observed between 85.9 and 102.9 °C in the DSC curves corresponds to the melting of crystalline drug within the fibers. FTIR confirmed the presence of API in the fibers, as well as interactions between the API molecules and PCL. SANS data clearly demonstrate the formation of drug aggregates within the polymer matrix. Upon contact with water, these aggregates evolve into larger drug-rich domains, accompanied by the development of water-filled pores as the APIs dissolve. The size of these domains increases with API loading. In vitro release studies showed that fibers with 10% loading exhibited a more sustained release profile, whereas those with 30% loading displayed a pronounced initial burst followed by a slower release.

## Supplementary Material



## Notes

^1^H NMR spectra confirming the synthesis of aspirin-D; simultaneous XRD–DSC data for pure aspirin; plots of the cumulative mass of drug released from electrospun PCL fibers with different drug loading; and correlations of cumulative drug release at 8 h with fiber diameter and the SANS-derived correlation length (PDF)
