# Supplementary material for: Probing Nanoscale Features of Electrospun Amorphous Solid Dispersions Using Advanced Biophysical Analysis
Source: Mol Pharm. 2026 Aug 1;23(9):4596–610. doi: 10.1021/acs.molpharmaceut.6c00342 (PMC13556136; doi:10.1021/acs.molpharmaceut.6c00342)
Supplement: Supplementary file 1 [file mp6c00342_si_001.pdf]

Supporting information for:

Probing nanoscale features of electrospun amorphous solid dispersions using advanced biophysical analysis

Luyao Wang[1], Abhishek Rajbanshi[1,2], Najet Mahmoudi[3], Evangelia Tsolaki[1], Thomas Zillhardt[4], Eleanor Hilton[1], Michael T. Cook[1]*, Gareth R. Williams[1]*

[1]UCL School of Pharmacy, University College London, 29-39 Brunswick Square, London WC1N 1AX, UK

[2] Implexis Ltd., 37-63 Southampton Row, London, WC1B 4DR, UK

[3]ISIS Pulsed Neutron and Muon Source, Rutherford Appleton Laboratory, Didcot OX11 0QX, UK

[4]Diamond Light Source, Harwell Science and Innovation Campus, Fermi Ave, Didcot OX11 0DE, UK

* Authors for correspondence. Email: michael.t.cook@ucl.ac.uk (MTC); g.williams@ucl.ac.uk (GRW)

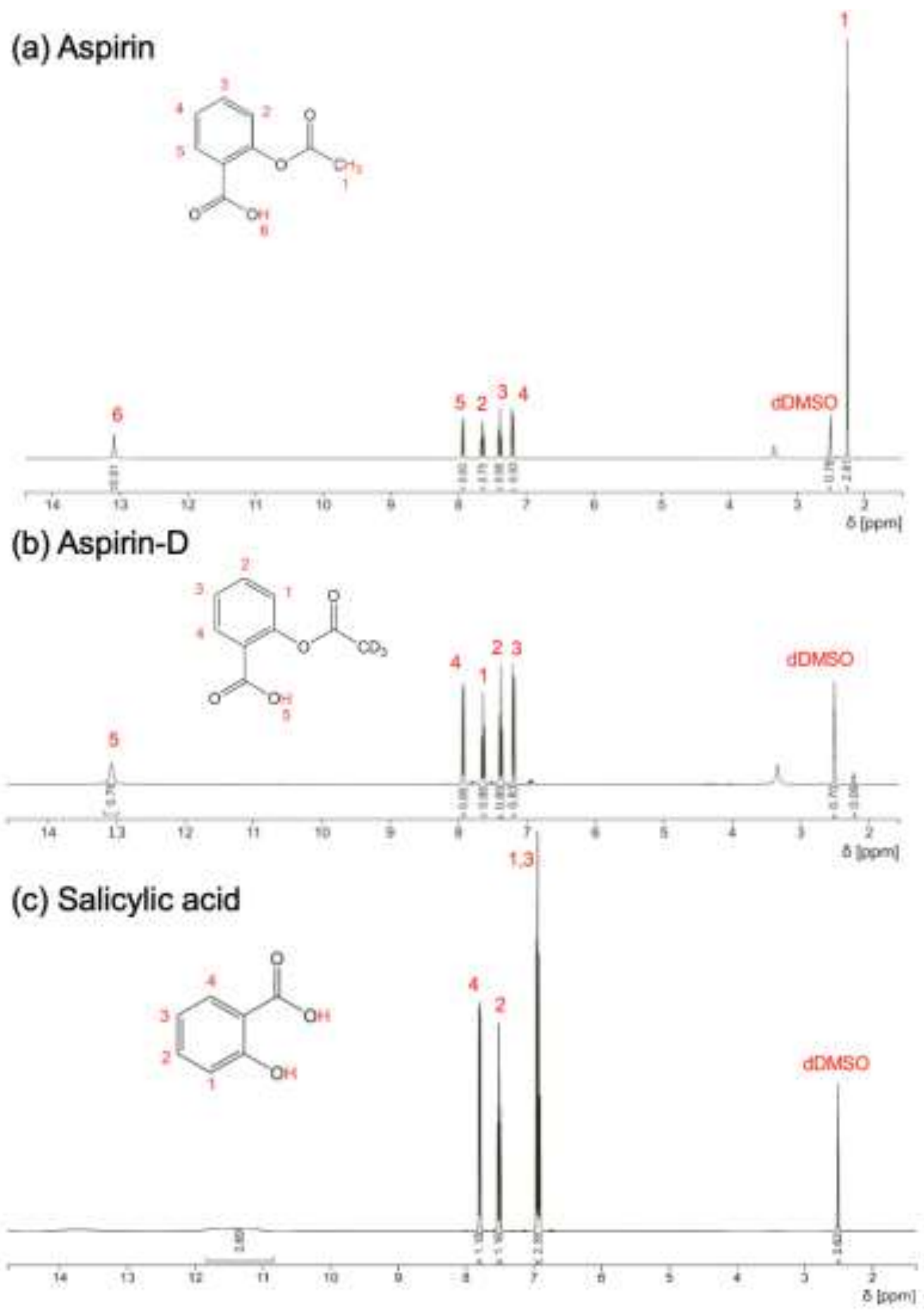

Figure S1. 400Hz ^{1}H nuclear magnetic resonance (NMR) spectra of: (a) aspirin, (b) aspirin-D and (c) salicylic acid. Small peaks at ca. 3.3 ppm in (a) and (b) correspond to residual water.

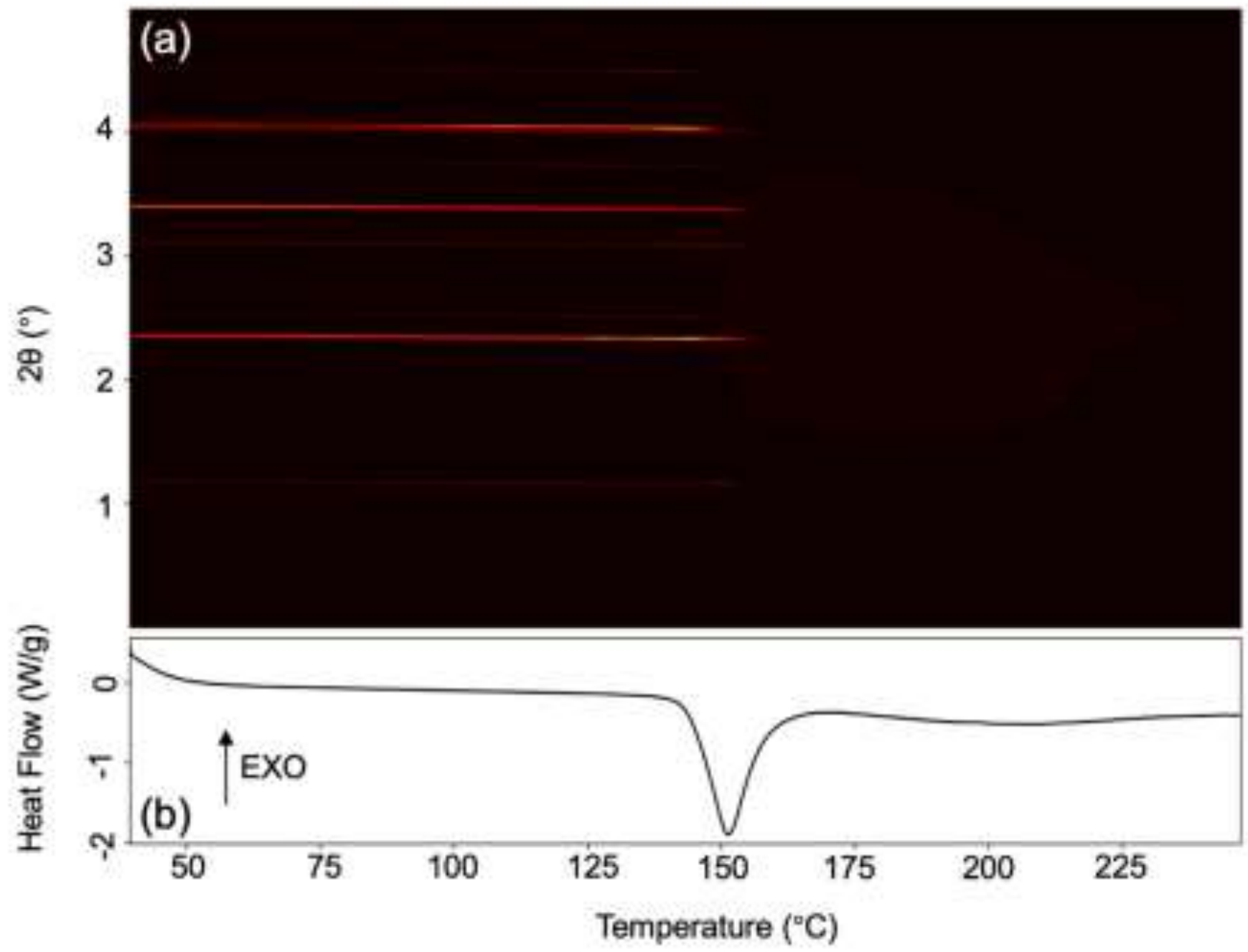

Figure S2. XRD–DSC data for pure aspirin, showing (a) XRD and (b) DSC data.

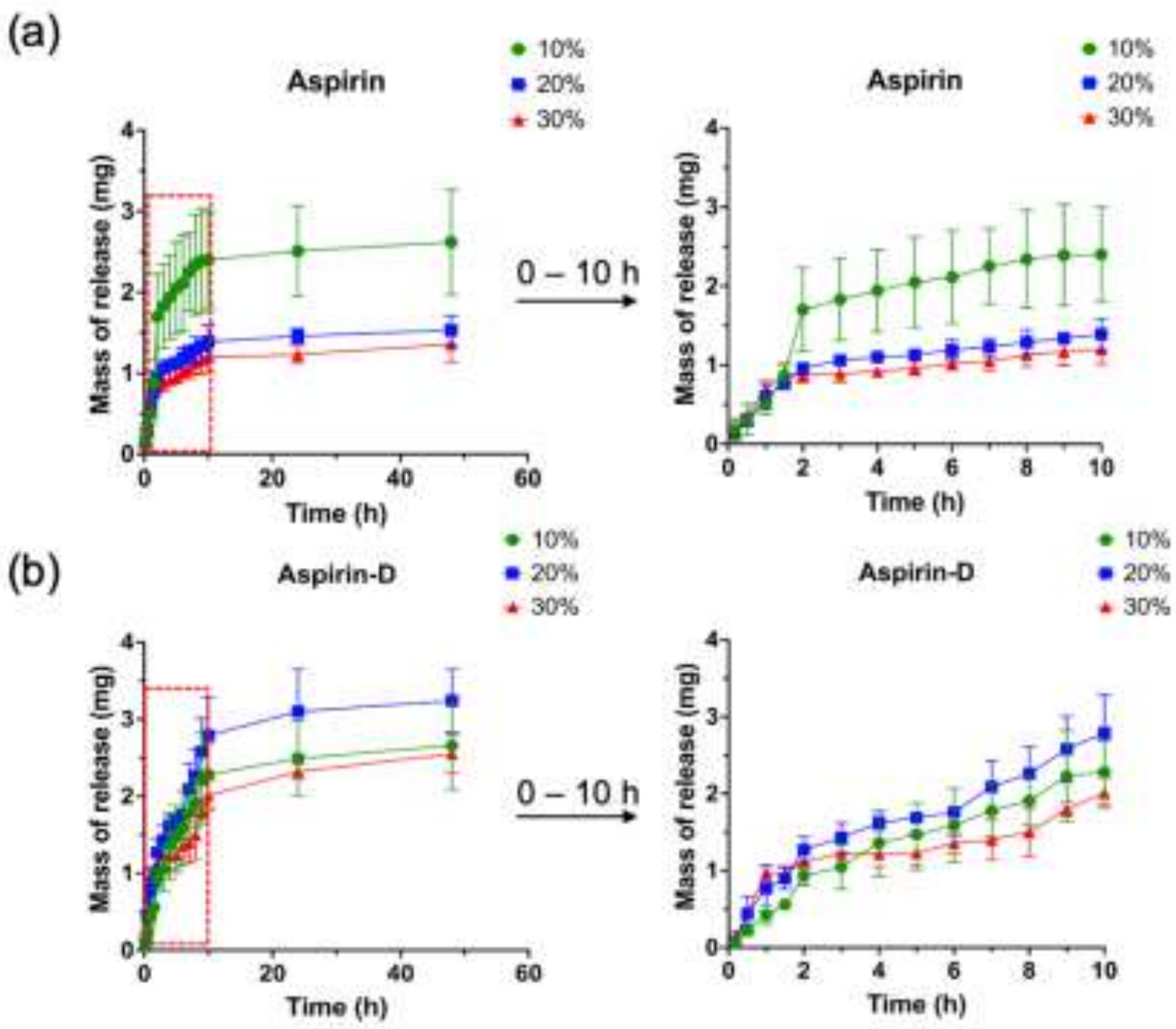

Figure S3. The cumulative mass of drug released from electrospun PCL fibers with different loadings: (a) aspirin and (b) aspirin-D. All data are shown as mean ± SD.

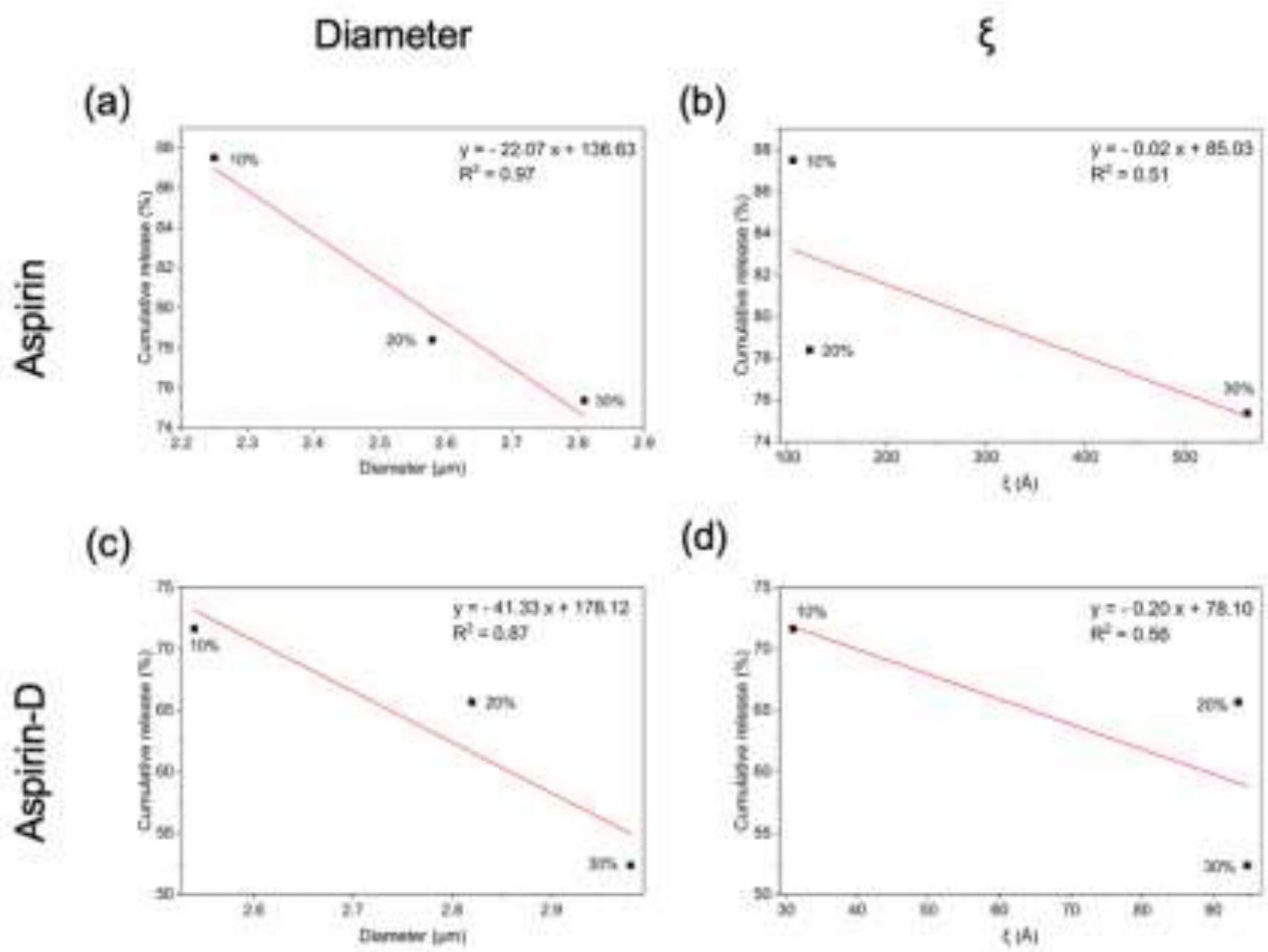

Figure S4. Correlation between cumulative drug release at 8 h and fiber diameters or SANS-derived correlation length, ξ. The cumulative percentage release at 8 h was plotted against the averaged fiber diameter for (a) aspirin and (c) aspirin-D-loaded fibers, and against ξ for (b) aspirin-loaded and (d) aspirin-D-loaded fibers. Linear regression lines are shown together with the corresponding regression equations and R^2 values. Each data point represents a formulation with a different drug loading. These trends are interpreted qualitatively due to the limited number of formulations.
